# Does in utero HIV exposure and the early nutritional environment influence infant development and immune outcomes? Findings from a pilot study in Pretoria, South Africa

**DOI:** 10.1186/s40814-020-00725-8

**Published:** 2020-12-11

**Authors:** Marina White, Ute D. Feucht, Eleanor Duffley, Felicia Molokoane, Chrisna Durandt, Edana Cassol, Theresa Rossouw, Kristin L. Connor

**Affiliations:** 1 Department of Health Sciences, Carleton University, Ottawa, ON K1S 5B6 Canada; 2 Paediatrics, University of Pretoria, Pretoria, GP 0002 South Africa; 3 Research Centre for Maternal, Fetal, Newborn & Child Health Care Strategies, University of Pretoria, Pretoria, GP 0002 South Africa; 4 Maternal and Infant Health Care Strategies Unit, South African Medical Council, Pretoria, South Africa; 5 Obstetrics and Gynaecology, University of Pretoria, Pretoria, GP 0002 South Africa; 6 South African Medical Research Council Extramural Unit for Stem Cell Research and Therapy, University of Pretoria, Pretoria, GP 0002 South Africa; 7 Institute for Cellular and Molecular Medicine, Department of Immunology, University of Pretoria, Pretoria, GP 0002 South Africa

**Keywords:** Pilot study, HIV, Neurodevelopment, Inflammation, Growth, Nutrition, Food security

## Abstract

**Background:**

As mother-to-child transmission of HIV decreases, and the population of infants who are born HIV-exposed, but uninfected (HEU) continues to rise, there is a growing need to understand the development and health outcomes of infants who are HEU to ensure that they have the healthiest start to life.

**Methods:**

In a prospective cohort pilot study at Kalafong Hospital, Pretoria, South Africa, we aimed to determine if we could recruit new mothers living with HIV on antiretrovirals (ART; *n* = 20) and not on ART (*n* = 20) and new mothers without HIV (*n* = 20) through our clinics to study the effects of HEU on growth and immune- and neurodevelopment in infants in early life, and test the hypothesis that infants who were HEU would have poorer health outcomes compared to infants who were HIV-unexposed, uninfected (HUU). We also undertook exploratory analyses to investigate relationships between the early nutritional environment, food insecurity and infant development. Infant growth, neurodevelopment (Guide for Monitoring Child Development [GMCD]) and levels of monocyte subsets (CD14, CD16 and CCR2 expression [flow cytometry]) were measured in infants at birth and 12 weeks (range 8–16 weeks).

**Results:**

We recruited 33 women living with HIV on ART and 22 women living without HIV within 4 days of delivery from June to December 2016. Twenty-one women living with HIV and 10 without HIV returned for a follow-up appointment at 12 weeks postpartum. The high mobility of this population presented major challenges to participant retention. Preliminary analyses revealed lower head circumference and elevated CCR2+ (% and median fluorescence intensity) on monocytes at birth among infants who were HEU compared to HUU. Maternal reports of food insecurity were associated with lower maternal nutrient intakes at 12 weeks postpartum and increased risk of stunting at birth for infants who were HEU, but not infants who were HUU.

**Conclusions:**

Our small feasibility pilot study suggests that HEU may adversely affect infant development, and further, infants who are HEU may be even more vulnerable to the programming effects of suboptimal nutrition in utero and postnatally. This pilot and preliminary analyses have been used to inform our research questions and protocol in our ongoing, full-scale study.

## Key messages regarding feasibility

- We conducted a prospective cohort pilot feasibility study in Pretoria, South Africa, to determine if we could recruit women through our clinics to study the effects of exposure to maternal HIV infection in utero and during the breastfeeding period on growth and immune- and neurodevelopment in infants uninfected with HIV in early life.
- While our study was able to capture information on infant growth, immune function and neurodevelopment at two time points within the same infant population, the high mobility of individuals from communities in this area presented challenges to participant retention and follow-up.
- In an aim to improve participant retention, in our ongoing, full-scale prospective pregnancy and birth cohort study at Kalafong Hospital, Ward-based Primary Health Care Outreach teams have been employed to trace and contact women who miss a follow-up appointment.

## Background

Maternal HIV infection profoundly affects maternal physiology and pregnancy outcomes. Annually, ~ 1.3 million women living with HIV become pregnant [1, 2], and HIV infection in pregnancy is associated with an increased risk of experiencing an adverse pregnancy outcome [3], including preterm birth and maternal mortality [4, 5]. Importantly, pregnancy and the postpartum period are two key times when fundamental structures in the fetus, such as the brain, undergo rapid development and are especially vulnerable to inflammatory or infectious challenges [6]. Thus, exposure to maternal HIV infection during these critical periods of development may have a lasting impact on the fetus and infant, including its brain development and function [7].

Infants who are HIV-exposed and infected (HEI) show poorer motor, cognitive, language and behavioural outcomes compared to controls as early as 3 months of age [8–10]. Importantly, global coverage of ART is increasing, reaching 80% of pregnant and breastfeeding women living with HIV in 2017 [11]. This is true in South Africa, which faces the largest burden of HIV globally and where an estimated 30% of pregnant women are living with HIV [12]. It is estimated that in 2018, 87% of women who were pregnant and living with HIV in South Africa had access to ART [13] and the rate of mother-to-child HIV transmission (MTCT) was below 2% at birth [12]. Thus, as a result of declining MTCT, the number of infants being born who are HIV-exposed (in utero and during breastfeeding) but *uninfected* (HEU) is rising [14].

Importantly, the extent to which HEU influences infant development is poorly understood, although evidence suggests that infants who are HEU have persistently altered motor and cognitive development [15–17], albeit to a lesser extent than infants who are HEI. Further, in adults with HIV, markers of monocyte activation and altered frequencies of monocyte subsets are among some of the best predictors of non-AIDS-associated co-morbid diseases [18, 19] and associate with increased neuro- and peripheral inflammation [20, 21]. Infants who are perinatally infected with HIV have increased monocyte activation between 4 and 15 weeks postpartum [22] relative to infants who are HEU. However, it is less clear whether the distribution of monocyte subsets and their migratory potential are altered in infants who are HEU compared to infants who are HIV-unexposed, uninfected (HUU). Whether or not these alterations in HEU could explain some of the adverse neurodevelopmental and growth outcomes recorded in infants who are HEU is not well understood.

We conducted a prospective cohort pilot feasibility study in Pretoria, South Africa, to determine if we could recruit women through our clinics to study the effects of HEU on growth and immune- and neurodevelopment in infants in early life. Our first objectives were to test the feasibility of our research protocol and study design and to identify barriers to long-term follow-up with mother-infant dyads. Our second objective was to perform exploratory analyses to test the hypothesis that infants who were HEU would have poorer growth and neurodevelopment and alterations in monocyte subsets compared to infants who were HUU.

Lastly, as South Africa reports high rates of food and nutrition insecurity [23], and the role of early life nutrition in infant health and development is well established [24–26], it is also critical to understand how the early nutritional environment interacts with infectious exposures to influence developmental trajectories in infants who are HEU. Thus, our third objective was to explore the food security circumstances and dietary intakes of the study participants and relate these to our outcome measures. Together, objectives two and three were intended to inform the planning of our aims and analyses for a full-scale, future study.

## Methods

### Design, aims and setting

This pilot feasibility study was an observational, prospective clinical cohort study that took place at the obstetric unit at Kalafong Provincial Tertiary Hospital. We aimed to recruit new mothers living with HIV on antiretrovirals (ART; *n* = 20) and not on ART (*n* = 20) and new mothers without HIV (*n* = 20) and their infants after delivery and follow them up in the early postpartum (PP) period, approximately 8–16 weeks after birth.

### Ethics

This study was approved by the Research Ethics Committee of the Faculty of Health Sciences of the University of Pretoria (185-2016) and the Carleton University Research Ethics Board (108870).

### Participant recruitment and eligibility

Eligible women were identified by a research nurse after delivery. Exclusion criteria included caesarean section delivery, pregnancy complications (pre-gestational diabetes or gestational diabetes mellitus), multiple gestations, or antibiotic exposure during labour or delivery and/or the postpartum period. Women were also ineligible to participate if they were from other regions and would find it difficult to come back for follow-up. All infants exposed to HIV were tested for infection at birth and 12 weeks postpartum. As we were interested in exploring the effects of HIV exposure without infection on infant growth, neurodevelopment, and immune outcomes, if an infant was determined to have HIV, the mother-infant dyad was subsequently excluded from the study analyses.

### Data collection

#### Maternal pregnancy and postnatal environment data

After delivery, a retrospective medical chart review was conducted to extract antenatal data. This included maternal characteristics (age at conception, parity, gravidity, smoking status, weight during pregnancy), medication use during pregnancy (including antibiotic exposure), illness/infections during pregnancy and pregnancy outcomes (gestation length). At the postpartum follow-up visit, mothers completed a questionnaire to assess breastfeeding practices, maternal lifestyle factors (including alcohol and smoking) and nutrition (including vitamin supplements, food security and a 24-h dietary recall). If any visits to clinics or hospitals occurred between birth and the follow-up visit, the patient-retained child health record (Road to Health Booklet [27]) of the infant was examined to extract data on infant weight, history of illness and medication use.

#### Infant outcomes at birth and 12 weeks postpartum

Infant weight, length and abdominal and head circumference were measured at birth and 12 weeks postpartum. Apgar score at 1 and 5 min was obtained. Infant anthropometry was age- and sex-standardised using the World Health Organization (WHO) growth standards (WHO Anthro software [v 3.2.2, January 2011]) [28]. A brain weight estimate was calculated using an equation derived by the National Institute of Neurological and Communicative Disorders and Stroke’s Collaborative Perinatal Project [29]: *brain weight (g) = 0.037 × head circumference (cm)*^*2.57*^. The brain weight estimate was used to calculate the infant brain-to-body weight ratio (BBR) [30]: *BBR = 100 × (brain weight estimate [g])/(birth weight [g])*. Weight gain from birth to 12 weeks postpartum (kg/day) was calculated using the weight of an infant at birth and follow-up and the days alive since birth at follow-up: *(weight at 12 weeks postpartum [kg] − weight at birth [kg])/number of days alive*.

#### Infant monocyte subsets

All infants who were HEU underwent a blood draw at birth and again at 12 weeks for HIV testing. Blood from this routine draw was obtained within 4 days of birth and again at 12 weeks and used to quantify the surface markers CD14, CD16 and CCR2 for monocyte subset identification [31]. Using the Gallios flow cytometer (3 laser, 10 colour configuration; Beckman Coulter, Miami, FL, USA), CD14 expression on PBMCs and CD16 and CCR2 expression (% and median fluorescence intensity [MFI]) on monocyte subsets was evaluated within 4 days of birth and at 12 weeks of age. The Kaluza V1.0 Acquisition software (Beckman Coulter, Miami, FL, USA) was used for data acquisition, and post-acquisition data analysis was performed using Kaluza Analysis software (version V3.1; Beckman Coulter, Miami, FL, USA). Flow-Check Pro fluorosphere was acquired daily prior to sample analysis to ensure optimal laser alignment and instrument performance. Single colour staining tubes (i.e. sample stained with individual monoclonal antibodies) were used to set up the protocol and calculate the colour compensation values. After setup and protocol/template verification, the instrument settings (voltages, gains, threshold and colour compensation settings) were kept the same throughout the study. The reagent list, including lasers and detectors used, and compensation matrix are presented in Supplementary tables S1 and S2, and the analysis approach and gating strategy are described in Supplementary figures S1 and S2. Classical (CD14^++^CD16^−^), intermediate (CD14^++^CD16^+^) and non-classical (CD14^+^CD16^+^) monocyte subsets were identified [32].

#### Infant neurodevelopment

The Guide for Monitoring Child Development (GMCD) [33] assesses expressive and receptive language, play activities, relating and response behaviour, and fine and large movement. The GMCD was developed for use in low- and middle-income countries to assess infants from 1 to 24 months postpartum, and involves the researcher asking the child’s caregiver a series of open-ended questions relating to the child’s development. An assessment for each infant was carried out once between 8 and 16 weeks postpartum. Infants who were 1–3 months of age (1 month to 2 months and 30 days) were assessed on milestones listed in the 1–3-month category, and infants who were 3–5 months (3 months +1 day to 4 months + 30 days) were assessed for milestones listed in both the 1–3- and 3–5-month columns. Infants who were premature (< 37 weeks) were age-corrected to term. The GMCD has been standardised and validated for international use in a sample of approximately 12,000 children from 4 diverse countries, namely South Africa, Argentina, India and Turkey [34]. The proportion of infants having attained all milestones (compared to not having attained all milestones) [35] in their age category (1–3 months or 3–5 months) was quantified.

#### Maternal reports of food security, dietary recall and infant feeding patterns

A questionnaire was developed to collect maternal reports of food security. Mothers were asked if, in the past 12 months, the following were ‘often true’, ‘sometimes true’ or ‘never true’: (1) They and other household members worried that food would run out before they got money to buy more; (2) the food that they and other household members bought just did not last, and there was not any money to get more; and (3) they and other household members could not afford to eat balanced meals. For the purpose of exploratory analyses and due to the small sample size of the pilot, maternal reports of ‘often true’ and ‘sometimes true’ were grouped together for analyses as ‘experiences food insecurity’ and compared with ‘never true’ responses.

Maternal dietary recall data collected a detailed account of all food and drink consumed in the day prior to the follow-up appointment. Dietary recall data were analysed using FoodFinder3 [36], a dietary analysis software programme developed by the South African Medical Research Council, specific to the nutrient composition of foods in South Africa. The estimated average requirements (EARs) and tolerable upper levels (TULs) for available nutrients from the Institute of Medicine Dietary Reference Intakes were used to evaluate the nutritional adequacy of reported maternal diets [37]. These reference intakes have been used previously to evaluate diet composition in various South African cohorts [38]. A dietary diversity score (DDS) was calculated as an additional measure of diet quality using nine food groups ((1) cereals, roots and tubers; (2) vegetables and fruits rich in vitamin A; (3) other fruits; (4) other vegetables; (5) legumes; (6) ,eat, poultry and fish; (7) dairy; (8) eggs; and (9) fats and oils) as previously validated and described in South African cohorts [39, 40]. Each food group was only counted once.

At follow-up, mothers reported whether they were, or had ever, exclusively breastfed their infants. If the infants were currently receiving formula, the mothers provided the age at which formula had been introduced.

### Statistical analyses

Data were analysed using JMP 14.0. One-way analysis of variance (ANOVA to test equality of means for normal data with equal variance, Kruskal-Wallis/Wilcoxon test to test equality of medians for non-parametric data or Welch’s test to test equality of means for normal data with unequal variance) and standard least squares linear regression (ANCOVA) models were used to explore (1) infant anthropometry at birth and 12 weeks postpartum; (2) Apgar scores (1 and 5 min); (3) levels of total monocytes, monocyte subsets and CCR2 expression assessed within 4 days of birth and at 12 weeks postpartum; and (4) number of GMCD milestones attained for infants who were HEU compared to infants who were HUU. Maternal age and weight at delivery, and infant gestational age at birth, sex, and age (days) at their follow-up appointment were included as covariables in the adjusted analysis. Hedge’s *g* with 95% confidence intervals (CI) and omega squared (*ω*^2^) were used to describe the standardised mean difference of effect for the ANCOVA models, and the proportion of the variance in response values that is explained by the model, respectively. We also explored maternal dietary intake nutrient levels for mothers living with and without HIV and possible relationships between household food insecurity and infant outcomes through comparisons of infant outcomes at birth and 12 weeks for infants whose mothers reported on food security. Data for infant outcomes below are presented as unadjusted means (SD) or medians (IQR) with *p* value from ANCOVA (*p* < 0.05).

We also explored associations between (1) attainment of all age-appropriate GMCD milestones and (2) stunting at birth or 12 weeks of age for infants who were HEU or HUU, as well as relationships between maternal reports of food insecurity and (1) stunting at birth or 12 weeks of age, (2) attainment of all age-appropriate GMCD milestones and (3) exclusively breastfeeding at follow-up. These data are presented below as relative risk (RR; 95% CI) and absolute risk difference (ARD; 95% CI) with *p* value from Fisher’s exact test (2-tail).

To determine whether or not the study cohort at follow-up was representative of the cohort at birth, one-way analysis of variance compared all outcomes at birth for infants who were, versus were not, present at follow-up, within the groups of infants who were HEU and HUU. Data below that compare outcomes in infants who were lost to follow-up to those who were not are presented as unadjusted means (SD) or medians (IQR) and *p* values (*p* < 0.05).

## Results

### Recruitment and participant study groups

**Fig. 1:**
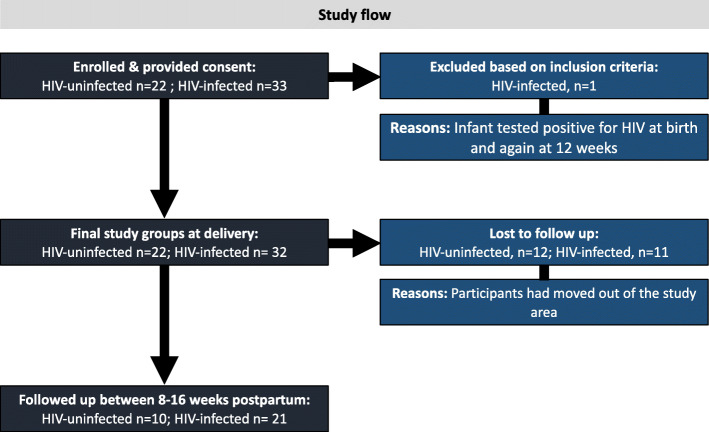
Study flow diagram. Thirty-three women living with HIV on ART and 22 women living without HIV were recruited within 4 days of delivery from June to December 2016. One infant whose mother was living with HIV tested positive for HIV at birth and again at 12 weeks and was excluded from the study analyses, making the final groups at delivery: HIV-uninfected, *n* = 22; HIV-infected, *n* = 32. 31.25% of women living with HIV and 54.5% of those living without HIV did not attend a follow-up appointment. Twenty-one women living with HIV and 10 without were followed up at one time point 12 weeks (range 8–16 weeks) postpartum. The high mobility of this population presented major challenges to participant retention

Study recruitment took place between June and December 2016. By March 2017, all follow-up data had been collected. An overview of the pilot study design, methods and participation is described in Fig. 1. At the end of recruitment, we recruited 55 women within 4 days of delivery: 33 living with HIV on ART and 22 living without HIV. Due to changed treatment policies, all women living with HIV were already on ART when they enrolled into the study. Secondly, the time set out for recruitment lapsed before we could enrol the planned numbers of women living without HIV. One infant whose mother was living with HIV tested positive for HIV at birth and again at 12 weeks. This mother-infant dyad was subsequently excluded from the study analyses, making the final groups at delivery: HIV-uninfected, *n* = 22; HIV-infected, *n* = 32. Attrition at follow-up was high, with 31.25% of women living with HIV and 54.5% of those living without HIV not attending a follow-up appointment. Thus, 21 women living with HIV and 10 without were followed up at one time point 12 weeks (range 8–16 weeks) postpartum. Multiple attempts were made to contact women who missed follow-up appointments via the phone numbers provided at recruitment. The high mobility of this population presented major challenges to participant retention.

### Maternal cohort characteristics

Maternal cohort characteristics are described in Table 1. There were no differences in maternal age, gravidity, parity, level of education or weight at delivery between mothers living with vs. without HIV. All mothers identified as non-cigarette smokers; however, one woman reported consuming snuff.

**Table 1:** Maternal cohort characteristics

Maternal characteristics	HIV uninfected (*n* = 22)	HIV infected (*n* = 32)	***p*** value
Weight at delivery (kg)	60.1 (55.0, 80.3)	60.4 (56.0, 69.0)	0.85
Age (years)	28.1 ± 7.31	30.8 ± 5.91	0.13
Gravidity (*n*)	2.00 (1.50, 4.00)	3.00 (2.00, 3.00)	0.22
Parity (*n*)	2.00 (1.00, 3.00)	2.00 (1.00, 3.00)	0.95
Level of education (*n*)			
Primary	1	6	
Secondary	17	24	
Post-secondary	4	1	
Not indicated	–	1	
Current ART (*n*)			
TDF, FTC, EFV	–	28	
AZT, 3TC, LPV/r	–	1	
None		3	

Data are means ± SD (ANOVA to test equality of means for normal data with equal variance) or median (IQR; Kruskal-Wallis/Wilcoxon test to test equality of medians for non-parametric data)

*TDF* tenofovir, *FTC* emtricitabine, *EFV* efavirenz, *AZT* azidothymidine, *3TC* lamivudine, *LPV/r* ritonavir-boosted lopinavir

### Infant outcomes at birth

#### Infant birth outcomes and demographics

Infant cohort characteristics are presented in Table 2. Among infants who were HUU, 45.5% were male, while 34.4% of infants who were HEU were male. Preterm birth (< 37 weeks) occurred in 5/54 (9.26%) pregnancies. Two preterm infants were HUU, born at 36 and 35 weeks, and three were HEU, with two born at 36 weeks and one at 35 weeks.

**Table 2:** Infant cohort characteristics and anthropometric, immune and neurodevelopmental outcomes at birth and 8–16 weeks postpartum

	HIV-unexposed-uninfected	HIV-exposed-uninfected	***p*** value (unadjusted)	***p*** value (adjusted)	Hedge’s ***g*** or RR (95% CI)	***ω***^**2**^
**Infant outcomes at birth**						
	*n* = 22	*n* = 32				
Infant sex (% male)	45.5	34.4	0.57	–	–	–
Gestational age at delivery (weeks)	38.4 ± 1.56	38.7 ± 1.69	0.48	–	–	–
< 37 weeks GA (*n*)	2	3	1.00	–	–	–
< 37 weeks GA (weeks)	35.5 (35.0, 36.0)	36.0 (35.0, 36.0)	1.00	–	–	–
* Infant anthropometry*^1^						
Weight *z*-score	− 0.57 ± 1.15	− 0.76 ± 0.96	0.52	0.48	0.22 (− 0.38, 0.82)	0.00
Length *z*-score	− 1.33 (− 3.20, 0.45)	− 1.65 (− 2.33, − 0.94)	0.55	0.89	0.04 (− 0.57, 0.65)	0.00
BMI *z*-score	− 0.15 (− 1.76, 1.80)	0.09 (− 0.81, 0.97)	0.89	0.39	0.27 (− 0.35, 0.88)	0.00
Head circumference *z*-score	− 0.33 ± 1.19	− 1.58 ± 1.22	< 0.001	< 0.001	1.15 (0.50, 1.80)	0.17
Brain-to-body weight ratio	10.2 (9.77, 11.3)	9.60 (8.88, 10.4)	0.06	0.05	0.62 (− 0.001, 1.24)	0.06
Stunting at birth^2^ (*n* [%])	7 (33.3)	14 (43.8)	0.57	–	1.31 (0.64, 2.70)	–
* Apgar score*						
1 min	9.00 (8.00, 9.00)	9.00 (8.00, 9.00)	0.96	0.43	0.24 (− 0.36, 0.85)	0.00
5 min	9.00 (9.00, 10.0)	9.00 (9.00, 9.00)	0.79	0.95	0.02 (− 0.59, 0.62)	0.00
* Immune measures*	*n* = 21	*n* = 32				
Total CD14+	10.5 (8.19, 12.2)	8.76 (6.23, 12.0)	0.10	0.08	0.56 (− 0.06, 1.19)	0.05
%CCR2-positive CD14+ monocytes	82.9 ± 6.91	86.6 ± 4.85	0.03	0.09	0.55 (− 0.08, 1.17)	0.04
Monocyte sub-populations (%)						
Classical (CD14++/CD16−)	67.8 ± 13.7	70.8 ± 13.3	0.44	0.50	0.21 (− 0.40, 0.83)	0.00
Intermediate (CD14++/CD16+)	14.7 (9.83, 21.2)	12.9 (9.35, 17.0)	0.14	0.36	0.29 (− 0.33, 0.90)	0.00
Non-classical (CD14+/CD16+)	8.40 (4.26, 15.4)	8.44 (4.17, 13.0)	0.74	0.97	0.01 (− 0.60, 0.62)	0.00
%CCR2-positive monocyte sub-populations						
Classical (CD14++/CD16−)	94.7 (92.2, 97.2)	97.3 (94.6, 98.4)	0.02	0.01	0.84 (0.20, 1.47)	0.11
Intermediate (CD14++/CD16+)	60.4 (51.4, 71.9)	68.2 (62.3, 80.9)	0.07	0.25	0.37 (− 0.26, 1.00)	0.01
Non-classical (CD14+/CD16+)	8.05 (4.00, 15.4)	6.88 (3.55, 15.1)	0.73	0.93	0.03 (− 0.59, 0.64)	0.00
CCR2 MFI on monocyte sub-populations						
Classical (CD14++/CD16−)	5.25 (4.49, 6.63)	6.29 (5.59, 6.81)	0.04	0.08	0.57 (− 0.06, 1.19)	0.05
Intermediate (CD14++/CD16+)	2.33 (2.16, 2.65)	2.87 (2.34, 3.48)	0.003	0.04	0.65 (0.02, 1.27)	0.06
Non-classical (CD14+/CD16+)	1.65 (1.55, 1.92)	1.70 (1.49, 2.22)	0.52	0.72	0.12 (− 0.50, 0.73)	0.00
**Infant outcomes at 12 weeks postpartum**						
	*n* = 10	*n* = 21				
Infant sex (% male)	50.0	33.3	0.45	–	–	–
Infant age at follow-up (weeks)	10.4 (10.1, 12.1)	12.0 (10.1, 13.3)	0.25	–	–	–
EBF at follow-up (*n* [%])	4 (40.0)	14 (81.0)	0.25	–	1.67 (0.74, 3.77)	–
* Infant anthropometry*^1^						
Head circumference *z*-score	− 0.73 (1.40, − 0.58)	− 1.47 (− 2.17, − 0.97)	0.03	0.66	0.20 (− 0.70, 1.10)	0.00
Weight *z*-score	0.12 (− 1.02, 0.56)	− 0.46 (− 0.93, 0.02)	0.24	0.67	0.20 (-0.72, 1.11)	0.00
Length *z*-score	− 0.95 (− 2.74, − 0.27)	− 1.18 (− 1.69, − 0.62)	0.85	0.67	0.20 (− 0.71, 1.09)	0.00
BMI *z*-score	0.44 ± 1.92	0.19 ± 1.12	0.65	0.76	0.15 (− 0.77, 1.06)	0.00
Brain-to-body weight ratio	7.39 (6.67, 8.04)	7.59 (7.08, 7.83)	0.50	0.51	0.32 (− 0.63, 1.27)	0.00
Weight gain (birth to 12 weeks PP; kg/day)	0.04 ± 0.01	0.03 ± 0.01	0.44	0.45	0.36 (− 0.57, 1.27)	0.00
Stunting at 12 weeks postpartum^2^ (*n* [%])	3 (30.0)	2 (9.52)	0.30	–	0.32 (0.06, 1.61)	–
*Immune measures*	*n* = 10	*n* = 17				
Total CD14+	7.40 (5.69, 9.78)	7.16 (5.69, 8.98)	0.74	0.23	0.65 (− 0.42, 1.70)	0.03
%CCR2-positive CD14+ monocytes	80.3 (74.9, 85.3)	76.0 (63.5, 80.2)	0.13	0.08	0.99 (− 0.12, 2.06)	0.12
Monocyte sub-populations (%)						
Classical (CD14++/CD16−)	68.0 (67.5, 75.3)	64.1 (57.3, 72.9)	0.09	0.11	0.90 (− 0.19, 1.97)	0.08
Intermediate (CD14++/CD16+)	8.49 (6.61, 13.7)	12.6 (8.95, 22.3)	0.08	0.18	0.74 (− 0.34, 1.79)	0.04
Non-classical (CD14+/CD16+)	16.7 ± 4.94	19.4 ± 5.85	0.22	0.14	0.82 (− 0.27, 1.88)	0.06
%CCR2-positive monocyte sub-populations						
Classical (CD14++/CD16−)	93.3 (89.3, 96.6)	94.7 (93.5, 96.2)	0.47	0.62	0.27 (− 0.77, 1.30)	0.00
Intermediate (CD14++/CD16+)	44.8 ± 23.1	56.2 ± 12.2	0.17	0.64	0.25 (− 0.79, 1.28)	0.00
Non-classical (CD14+/CD16+)	2.14 (1.02, 3.49)	3.42 (1.70, 4.32)	0.26	0.55	0.33 (− 0.72, 1.36)	0.00
CCR2 MFI on monocyte sub-populations						
Classical (CD14++/CD16−)	4.67 (3.83, 6.04)	4.46 (4.03, 4.89)	0.25	0.08	1.03 (− 0.13, 2.17)	0.12
Intermediate (CD14++/CD16+)	2.19 (1.95, 3.14)	2.22 (1.89, 2.49)	0.50	0.02	1.45 (0.22, 2.64)	0.26
Non-classical (CD14+/CD16+)	1.55 (1.40, 2.52)	1.64 (1.40, 1.93)	0.85	0.31	0.58 (− 0.53, 1.68)	0.01
* Attainment of all 1–3-month GMCD milestones (n [%])*^3^	*n* = 8	*n* = 14				
Expressive language	7 (87.5)	13 (92.9)	0.36	–	1.14 (0.88, 1.49)	–
Receptive language	6 (75.0)	12 (85.7)	0.60	–	1.14 (0.73, 1.80)	–
Large movement	6 (75.0)	13 (92.9)	0.53	–	1.24 (0.81, 1.90)	–
Fine movement	8 (100)	14 (100)	–	–	–	–
Relating and response behaviour	7 (87.5)	13 (92.9)	1.00	–	1.06 (0.79, 1.43)	–
Play behaviour	5 (62.5)	13 (92.9)	0.12	–	1.49 (0.85, 2.59)	–

Data are presented means ± SD (ANOVA to test equality of means for normal data with equal variance) or median (IQR; Kruskal-Wallis/Wilcoxon test to test equality of medians for non-parametric data or Welch’s test to test equality of means for normal data with unequal variance) with *p* values from one-way analysis of variance and adjusted multiple variable regression models. Differences in proportions for infant sex, < 37 weeks gestational age, % EBF (exclusively breastfed) at follow-up and risk of stunting for HUU vs. HEU infants were assessed by Fisher’s exact test (2-tail). Hedge’s *g* and RR are reported as effect size estimates for continuous and categorical infant outcome data, respectively. Omega square (*ω*^2^) denotes variance accounted for by the model. Infant anthropometry was adjusted for maternal age, maternal weight and gestational age. BBR, Apgar score (1 and 5 min), immune measures at birth, and weight gain (birth to 12 weeks PP; kg/day) were adjusted for maternal age, maternal weight, gestational age and infant sex

*RR* relative risk, *PP* postpartum

^1^All infant anthropometric measures are standardised according to WHO child growth standards [28]

^2^Stunting is determined by <− 2 SD length-for-age standardised *z*-score according to WHO child growth standards [28]

^3^For fine movement milestones at 1–3 months, all infants in both groups met all milestones so no comparisons were made. There was only 1 HUU infant who was 3–5 months at follow-up, so no comparisons for GMCD 3–5 month milestones were made between the two groups

#### Anthropometric measures and Apgar scores

**Fig. 2:**
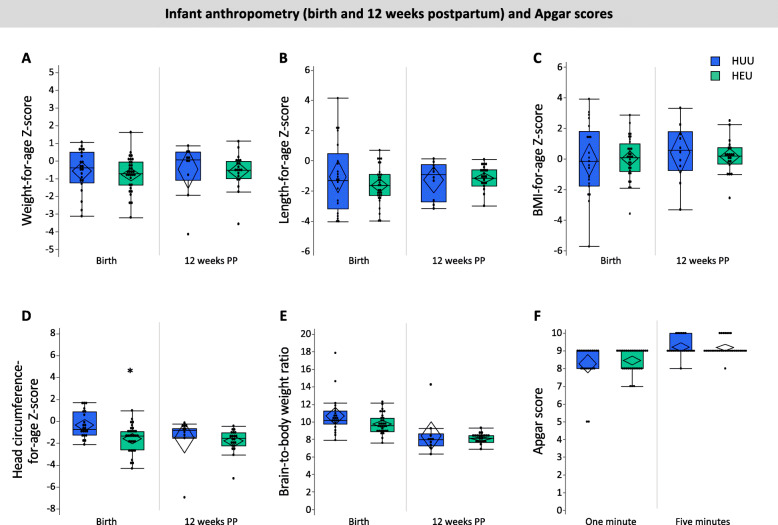
Relationships between in utero HIV exposure and infant anthropometry at birth and 12 weeks (**a**–**e**) and Apgar scores (**f**). Infants who were HEU (*n* = 32) had lower head circumference *Z*-scores at birth (**d**; *p* < 0.001) compared to infants who were HUU (*n* = 22). There were no other differences between infants who were HEU compared to HUU for anthropometry at birth or 12 weeks of age. Outlier box plots are measured anthropometry and Apgar scores (quartiles, median lines and 95% confidence diamonds, *p < 0.05 [ANCOVA]). HUU HIV-unexposed, uninfected; HEU HIV-exposed, uninfected; CI confidence interval

Exploratory analyses revealed lower head circumference-for-age *z*-scores (− 1.58 ± 1.22 vs. − 0.33 ± 1.19, *p* < 0.001; Fig. 2d, Table 2) at birth among infants who were HEU compared to HUU. There were no differences between infants who were HEU and HUU for BMI, length-for-age and weight-for-age *z*-scores, BBR at birth or Apgar scores (1 min, 5 min) (Fig. 2, Table 2).

#### Immune measures

**Fig. 3:**
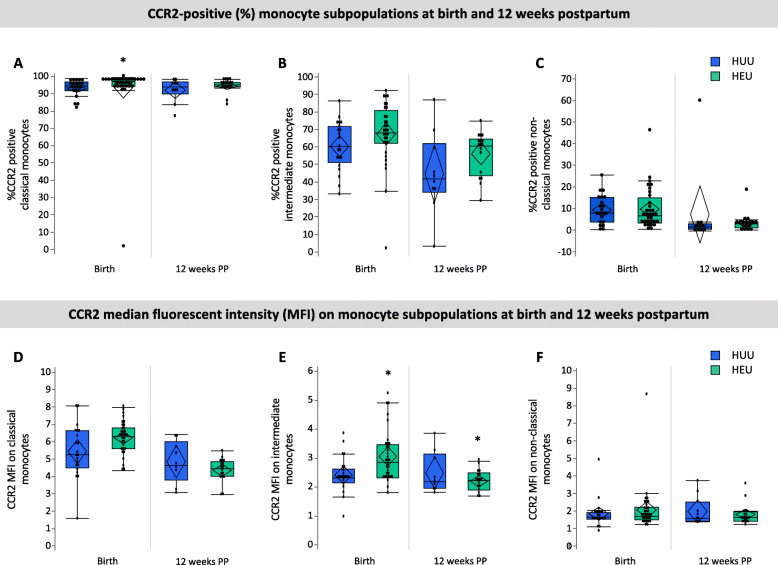
CCR2 expression by monocyte sub-populations within 4 days of birth and at 12 (± 4) weeks of age. Infants who were HEU (*n* = 32) had elevated %CCR2-positive classical monocytes (**a**; *p* = 0.01) and CCR2 expression (MFI) on intermediate monocytes at birth (**e**; *p* = 0.03) and 12 weeks (**e**; 0.04) compared to infants who were HUU (*n* = 21). Data are measured as proportion of monocyte sub-populations expressing CCR2 (%) and the average (median) levels of expression per cell (MFI) on monocyte sub-populations. Outlier box plots are quartiles, median lines and 95% confidence diamonds, *p < 0.05 [ANCOVA]. HUU HIV-unexposed, uninfected; HEU HIV-exposed, uninfected; MFI median fluorescent intensity (MFI); CI confidence interval

There were no differences between infants who were HEU vs. HUU in the relative frequency of total monocytes (% CD14+ PBMC), CCR2 expression by total CD14+ monocytes or the monocyte subsets (Table 2). Infants who were HEU had elevated %CCR2-positive classical monocytes (97.3 [94.6, 98.4] vs. 94.7 [92.2, 97.2], *p* = 0.01; Fig. 3a, Table 2) at birth. No differences were observed for %CCR2-positive intermediate- or non-classical monocytes between the two groups at birth (Fig. 3). Median fluorescence intensity (MFI) is indicative of the level of cell surface expression, and a higher CCR2 MFI was observed for intermediate (2.87 [2.34, 3.48] vs. 2.33 [2.16, 2.65], *p* = 0.04; Fig. 3e) at birth in infants who were HEU when compared to infants who were HUU. No significant differences in CCR2 expression levels were observed in classical or non-classical monocytes at birth in infants who were HEU compared to HUU (Fig. 2f, Table 2).

### Infant outcomes at 12 weeks postpartum

#### Demographics

At follow-up, 50.0% of infants who were HUU were male, while 33.3% of infants who were HEU were male (Table 2). There were no differences between infants who were HUU and HEU for age at follow-up.

#### Anthropometric measures

There were no differences between infants who were HEU and HUU for age- and sex-standardised anthropometric measures at follow-up (Fig. 2, Table 2), and no differences in weight gain from birth to 12 weeks postpartum (Table 2).

#### Immune measures

Infants who were HEU had elevated CCR2 MFI on intermediate monocytes compared to HUU at 12 weeks postpartum (2.22 [1.89, 2.49] vs. 2.19 [1.95, 3.14], *p* = 0.04; Fig. 3e), but not on classical or non-classical monocytes. There were no differences between infants who were HEU compared to HUU at 12 weeks postpartum for %CD14^+^ PMBCs, mean monocyte subsets or %CCR2 on all monocyte subsets (Table 2).

#### GMCD

**Fig. 4:**
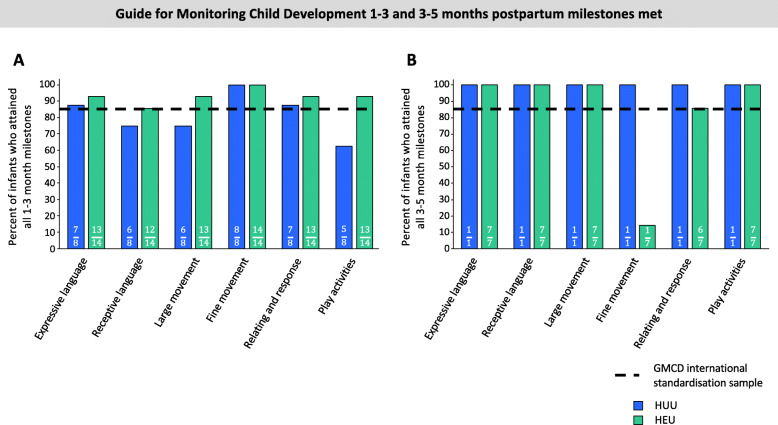
Relationships between in utero HIV exposure and attainment of GMCD neurodevelopmental milestones 1–3 months (**a**) and 3–5 months (**b**) of age. There was no detected difference in the proportion of infants who were HUU (*n* = 8) compared to HEU (*n* = 14) who attained GMCD milestones at 1–3 months of age ([*p* > 0.05], Fisher’s exact), and no comparisons between infants who were HUU vs. HEU were made for 3–5-month milestones met, given that only 1/10 infants who were HUU at follow-up was 3–5 months of age. All infants met milestones when comparing to the GMCD standardisation sample, except for infants who were HUU for 1–3-month receptive language, large movement and play activities milestones, and infants who were HEU for fine movement milestones at 3–5 months of age. Bar graphs are proportion (%) attaining all age-appropriate GMCD milestones. GMCD Guide for Monitoring Child Development; HUU HIV-unexposed, uninfected; HEU HIV-exposed, uninfected

The percentage of infants in each group (HUU compared to HEU) who had achieved all age-appropriate milestones at 1–3 or 3–5 months postpartum are presented (Fig. 4). One infant who was HUU did not undergo a GMCD developmental assessment at follow-up. Twenty-three of 31 (74.19%) of infants aged 8–16 weeks had a 1–3-month GMCD assessment. Of these infants, 15/23 (65.2%) were HEU. At 1–3 months, infants who were HUU had attained age-appropriate milestones for receptive language, large movement and play activity milestones in lower proportions (− 10.0%, − 10.0% and − 22.5%, respectively) than the GMCD international standardised sample (Fig. 4a). Infants who were HEU who were 1–3 months of age had attained all age-appropriate milestones in line with the GMCD international standardised sample for 1–3 months postpartum. We found no association between the HIV-exposure group and probability of attaining all age-appropriate milestones (compared to not attaining) in any GMCD neurodevelopmental theme at 1–3 months of age for infants who were HEU compared to HUU (Table 2). All infants who were 3–5 months at follow-up had attained all 1–3-month milestones for all themes, consistent with the GMCD international standardised sample.

A 3–5-month assessment was performed on the 8/31 (25.8%) infants at follow-up who were within this age range. Of these infants, 7/8 (87.5%) were HEU. Fewer infants at 3–5 months of age who were HEU had attained age-appropriate fine movement milestones in comparison to the GMCD international standardised sample (14.3% [*n* = 1/7] of HEU attained vs. 85% standardised sample, Fig. 4b). All of these infants who were HEU had attained all age-appropriate milestones for expressive language, receptive language, large movement and play activities, and met the international standardised sample proportion for relating and response behaviour (+ 0.70%). The one infant who was HUU and 3–5 months of age attained all developmental milestones.

### Differences between the cohort at birth and 12 weeks postpartum

Within the group of infants who were HUU, those who were not lost to follow-up had lower BMI at birth than those who were lost to follow-up (− 1.32 ± 2.47 vs. 0.90 ± 1.85, *p* = 0.03). There were no other differences for anthropometric, Apgar scores or immune measures at birth within the HUU or HEU infant groups, when comparing infants who were versus were not at follow-up.

### Exploring food security and maternal nutrition

#### Food security and nutrient intakes among women living with and without HIV

There were no differences between mothers living with and without HIV for the probability of reporting household food insecurity or DDS (Table 3). Mothers living with HIV had higher intakes of vitamin D (64.5 [42.0, 84.6] vs. 8.60 [0.38, 20.8], *p* = 0.002) and Se (51.7 [42.1, 73.7] vs. 12.6 [7.41, 34.4], *p* < .001) compared to mothers living without HIV (Table 3). Full data on absolute nutrient intake levels for mothers living with and without HIV from the 24-h dietary recall are presented in Supplementary table S3.

**Table 3:** Maternal reports of household food security and nutrient intakes from one 24-h dietary recall for mothers with and without HIV who attended follow-up

	Total, *N* = 31	HIV-uninfected (*n* = 10)	HIV-infected (*n* = 21)	***p*** value
**Household food security circumstances (*n*)**				
Maternal reports of worrying about food runout				0.28
Never occurs	14	3	11	
Occurs often/sometimes	17	7	10	
Maternal reports of experiencing about food runout				0.46
Never occurs	16	4	12	
Occurs often/sometimes	15	6	9	
Maternal reports of being unable to afford balanced meals				0.68
Never occurs	9	2	7	
Occurs often/sometimes	26	8	14	
**Maternal nutrient intakes**				
Dietary diversity score (/9)	4.00 (4.00, 6.00)	4.00 (3.00, 5.25)	4.00 (3.00, 6.00)	0.40
Dietary diversity score < 4 (n)	7	3	4	0.65
**Median intake (%) of TULs by mothers**				
*Minerals*				
Ca	5.48 (3.40, 9.56)	7.78 (4.09, 14.4)	5.28 (3.40, 7.48)	0.31
Fe	24.7 (16.7, 27.8)	25.0 (15.5, 30.1)	24.2 (16.4, 27.8)	0.95
Mg	52.9 (34.6, 81.1)	80.0 (33.0, 99.1)	48.0 (38.4, 64.0)	0.12
P	14.9 (10.8, 19.3)	18.1 (10.4, 21.4)	14.3 (10.5, 18.3)	0.84
Na	69.9 (48.2, 154)	92.4 (43.9, 171)	69.9 (47.8, 146)	0.82
Zn	20.9 (16.8, 29.3)	17.4 (15.2, 23.8)	23.7 (19.8, 29.9)	0.19
Cu	7.70 (5.50, 11.0)	11.4 (4.97, 13.4)	7.40 (5.20, 9.55)	0.23
Se	7.00 (4.15, 9.78)	1.86 (1.09, 5.08)	7.63 (6.21, 10.9)	< .001
Mn	12.7 (7.52, 17.6)	15.7 (6.64, 21.9)	10.2 (7.28, 16.2)	0.46
I	8.36 (4.09, 16.0)	8.27 (2.30, 16.1)	8.36 (5.86, 17.2)	0.46
*Vitamins*				
Vitamin A	14.1 (10.2, 23.8)	14.1 (11.1, 24.2)	14.3 (10.0, 23.5)	0.88
Niacin	44.0 (30.6, 55.7)	41.6 (29.1, 57.9)	46.6 (28.5, 55.9)	0.71
Vitamin B_6_	2.88 (1.60, 3.80)	2.61 (1.37, 5.19)	3.13 (1.72, 3.72)	0.60
Folic acid	25.9 (20.5, 31.9)	23.9 (18.3, 38.3)	27.8 (20.7, 31.2)	0.63
Vitamin C	1.15 (0.40, 3.65)	1.58 (0.11, 4.74)	1.10 (0.53, 3.13)	0.88
Vitamin D	4.29 (0.83, 7.86)	0.86 (0.04, 2.08)	6.45 (4.20, 8.46)	0.002
Vitamin E	0.68 (0.44, 1.19)	0.52 (0.33, 1.17)	0.80 (0.47, 1.33)	0.36
**Median intake (%) of EARs by mothers**				
*Macronutrients*				
Protein	66.6 (49.9, 90.4)	68.7 (60.6, 87.7)	65.5 (48.9, 99.5)	0.81
Carbohydrates	98.7 (63.6, 113)	81.8 (56.7, 81.8)	99.8 (77.7, 118)	0.41
*Vitamins*				
Vitamin A	47.0 (34.1 75.4)	46.9 (36.8, 77.6)	47.8 (33.4, 78.3)	0.88
Thiamin	90.0 (56.7, 111)	88.3 (53.5, 116)	90.0 (57.5, 118)	0.73
Riboflavin	55.4 (43.1, 74.6)	38.9 (24.4, 96.9)	58.5 (49.6, 74.2)	0.16
Niacin	119 (72.3, 150)	112 (69.4, 156)	125 (75.4, 150)	0.63
Vitamin B_6_	170 (88.7, 223)	139 (80.6, 306)	184 (97.4, 219)	0.54
Folate	54.2 (43.1, 70.9)	47.1 (39.9, 85.1)	61.8 (45.9, 69.2)	0.42
Vitamin B_12_	70.8 (62.5, 108)	68.8 (9.38, 102)	75.0 (66.7, 133)	0.24
Vitamin C	23.0 (8.00, 73.0)	31.5 (2.25, 92.7)	22.0 (10.5, 62.5)	0.92
Vitamin D	42.9 (8.30, 78.6)	8.60 (0.38, 20.8)	64.5 (42.0, 84.6)	0.002
Vitamin E	42.5 (27.4, 74.3)	32.7 (20.8, 73.1)	49.4 (29.5, 82.8)	0.36
*Minerals*				
Ca	17.1 (10.6, 29.9)	24.3 (12.7, 44.5)	16.5 (10.6, 23.4)	0.33
Fe	171 (115, 192)	168 (112, 192)	173 (106, 208)	0.88
Mg	71.3 (45.7, 111)	110 (44.0, 136)	63.4 (50.8, 87.1)	0.16
P	103 (68.3, 133)	125 (41.2, 147)	98.8 (69.5, 126)	0.94
Zn	78.9 (64.5, 113)	66.8 (47.4, 91.6)	91.0 (73.3, 115)	0.17
Cu	77.0 (52.8, 110)	112 (48.1, 127)	74.0 (50.4, 95.5)	0.31
Se	47.5 (28.1, 66.3)	12.6 (7.41, 34.4)	51.7 (42.1, 73.7)	< .001
I	44.0 (21.5, 80.9)	43.5 (12.1, 80.4)	44.0 (30.4, 90.4)	0.39

Data from mother-infant dyads that attended follow-up are presented as median (IQR) and *p* values are from one-way analysis of variance (Kruskal-Wallis/Wilcoxon test to test equality of medians for non-parametric data; ANOVA to test equality of means for normal data with equal variance). Dietary diversity score was calculated using nine food groups, only counting each food group once. Percent intake of TULs and EARs were calculated using the Institute of Medicine’s TULs for minerals and vitamins for lactating women 14–18, 19–30 or 31–50 years of age [37]. Iodised salt was assumed to be consumed by all mothers, as the majority of salt consumed in South Africa is iodised [61]

*TULs* tolerable upper levels, *EARs* estimated average requirements

#### Household food insecurity and diet quality

Full data on maternal reports of food insecurity and nutrient intakes is presented in Supplementary table S4. Overall, a large proportion of mothers were at risk of inadequate intake of macronutrients, vitamins and minerals, irrespective of reports of worrying about or experiencing food runout, or inability to afford balanced meals (Supplementary fig S3). Food insecurity was associated with an increased risk of inadequate intake (median %EAR met) of vitamin B_12_ among mothers who reported experiencing (66.7 [12.5, 91.7] vs. 89.6 [67.7, 144], *p* = 0.01) food runout, or an inability to afford balanced meals (66.7 [19.8, 96.9] vs. 108 [70.8, 150], *p* = 0.04) compared to mothers who did not (Supplementary fig S3). Of the mothers who reported worrying about or experiencing food runout, or inability to afford balanced meals, 76.5%, 86.7% and 81.8% were at risk for inadequate intake, respectively. Overall, very few mothers, irrespective of food insecurity reports, had intake of vitamins or minerals that was too high; however, TULs were exceeded for magnesium and sodium (Supplementary table S4). There were no relationships between reports of household food insecurity and DDS (Supplementary table S4).

#### Infant feeding patterns

**Fig. 5:**
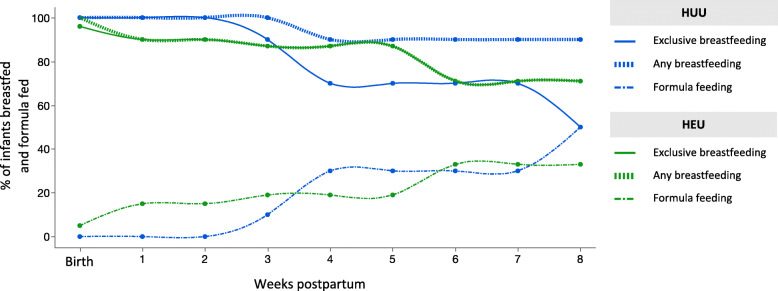
Feeding patterns from birth to 8 weeks postpartum for infants who were HUU and HEU. There were no differences in the likelihood of being exclusively breastfed (EBF) at 12 weeks of age for infants who were HUU (*n* = 10) compared to HEU (*n* = 21; [*p* > 0.05], Fisher’s exact 2-tail). Data on feeding practices were available for the whole cohort from birth to 8 weeks postpartum. Each point on the line represents the proportion (%) of infants who were HUU or HEU and were exclusively breastfed, were receiving any breastmilk or were formula fed at that time (weeks). HUU HIV-unexposed, uninfected; HEU HIV-exposed, uninfected

To explore the feeding patterns of infants who were HEU compared to HUU, we plotted the percentage of infants who were HEU or HUU and were breastfed (mixed and exclusive) or formula fed from birth to 8 weeks of age (Fig. 5). Feeding practices were only available until 8 weeks of age for the youngest infant at follow-up, so this was chosen at the cutoff to plot feeding patterns for the whole pilot cohort. At birth, exclusive breastfeeding (EBF) was initiated for all but one infant, who was HEU and received mixed feeds*.* At follow-up, slightly more infants who were HEU were still being exclusively breastfed compared to HUU; however, most infants who were HUU were still receiving some breastmilk (Fig. 5). There were no differences between infants who were HUU compared to HEU for the likelihood of EBF at follow-up (Table 2). Mothers who reported experiencing food runout (RR = 0.41 [0.19, 0.87], ARD = − 0.48 [− 0.78, − 0.17], *p* = 0.01) or were unable to afford balanced meals (RR = 0.51 [0.31, 0.85], ARD = − 0.43 [− 0.73, − 0.14], *p* = 0.045) were less likely than mothers who never experienced food runout, or were always able to afford balanced meals, to be EBF at follow-up, irrespective of maternal HIV status (Table 4).

**Table 4:** Anthropometry and Apgar scores at birth, and anthropometry, neurodevelopmental outcomes and breastfeeding practices at 12 weeks postpartum for infants exposed to food-insecure conditions compared to those who were not

**Do you worry about food runout?**						
	**No**	**Yes**	***p*** **value (unadjusted)**	***p*** **value (adjusted)**	**Hedge’s** ***g*** **or RR (95% CI)**	***ω***^**2**^
	*n* = 14	*n* = 17				
Gestational age at delivery (weeks)	38.8 ± 1.81	38.4 ± 1.66	0.49	–	–	–
EBF at 12 weeks PP (*n* [%])	11 (78.6)	7 (41.4)	0.07	–	–	–
* Infant anthropometry and Apgar at birth*^1^						
Head circumference *z*-score	− 1.49 ± 0.99	− 0.81 ± 1.46	0.15	0.35	0.37 (− 0.40, 1.14)	0.00
Weight *z*-score	− 0.59 ± 1.00	− 1.08 ± 1.10	0.22	0.20	0.51 (− 0.27, 1.29)	0.02
Length *z*-score	− 1.20 ± 1.41	− 1.05 ±. 2.10	0.82	0.35	0.38 (− 0.41, 1.16)	0.00
BMI *z*-score	− 0.02 ± 1.81	− 0.94 ± 1.64	0.16	0.59	0.22 (− 0.56, 1.00)	0.00
Brain-to-body weight ratio	9.65 (8.90, 10.3)	10.4 (9.70, 12.0)	0.05	0.17	0.56 (− 0.23, 1.34)	0.04
Stunting at birth^2^ (*n* [%])	3 (21.4)	8 (47.1)	0.26	–	2.04 (0.67, 6.21)	–
Apgar 1-min score	9.00 (8.75, 9.00)	8.00 (8.00, 9.00)	0.02	0.18	0.55 (− 0.25, 1.33)	0.03
Apgar 5-min score	10.0 (9.00, 10.0)	9.00 (9.00, 9.00)	0.02	0.01	1.19 (0.32, 2.03)	0.21
* Infant anthropometry in infants aged 12 weeks*^1^						
Head circumference *z*-score	− 1.47 (− 2.12, − 0.97)	− 1.07 (− 1.94, − 0.62)	0.36	0.54	0.25 (− 0.53, 1.01)	0.00
Weight *z*-score	− 0.08 (− 0.77, 0.23)	− 0.45 (− 1.26, 0.14)	0.45	0.22	0.52 (− 0.30, 1.32)	0.02
Length *z*-score	− 0.84 ± 0.64	− 1.52 ± 0.99	0.04	0.01	1.14 (0.29, 1.96)	0.17
BMI *z*-score	0.37 ± 0.96	0.19 ± 1.74	0.72	0.73	0.14 (− 0.66, 0.94)	0.00
Brain-to-body weight ratio	7.40 (6.84, 7.74)	7.61 (7.11, 7.91)	0.18	0.18	0.70 (− 0.32, 1.69)	0.04
Weight gain (birth to 12 weeks PP; kg/day)	0.03 ± 0.01	0.03 ± 0.01	0.49	0.34	0.41 (− 0.42, 1.22)	0.00
Stunting at 12 weeks postpartum^2^ (*n* [%])	1 (7.14)	4 (23.5)	0.35	–	3.29 (0.41, 26.2)	–
* Attainment of all 1-3 month GMCD milestones*^3^ *(n [%])*	*n* = 13	*n* = 9				
Expressive language	13 (100)	8 (88.9)	0.41	–	0.89 (0.71, 1.12)	–
Receptive language	11 (84.6)	7 (77.8)	1.00	–	0.92 (0.61, 1.40)	–
Large movement	13 (100)	6 (66.7)	0.06	–	0.67 (0.42, 1.06)	–
Fine movement	13 (100)	9 (100)	-	–	–	–
Relating and response behaviour	13 (100)	7 (77.8)	0.16	–	0.78 (0.55, 1.10)	–
Play behaviour	12 (92.3)	6 (66.7)	0.26	–	0.72 (0.44, 1.18)	–
**Do you experience food runout?**						
	**No**	**Yes**	***p*** **value (unadjusted)**	***p*** **value (adjusted)**	**Hedge’s** ***g*** **or RR (95% CI)**	***ω***^**2**^
	*n* = 16	*n* = 15				
Gestational age at delivery (weeks)	38.6 ± 1.79	38.5 ± 1.69	0.96	–	–	–
EBF at 12 weeks PP (*n* [%])	13 (81.3)	5 (33.3)	0.01	–	0.41 (0.19, 0.87)	–
* Infant anthropometry and Apgar at birth*^1^						
Head circumference *z*-score	− 1.39 ± 0.96	− 0.82 ± 1.56	0.23	0.50	0.28 (− 0.52, 1.07)	0.00
Weight *z*-score	− 0.59 ± 0.94	− 1.14 ± 1.15	0.16	0.14	0.62 (− 0.20, 1.42)	0.04
Length *z*-score	− 1.21 ± 1.36	− 1.02 ± 2.20	0.78	0.15	0.60 (− 0.22, 1.41)	0.04
BMI *z*-score	− 0.02 ± 1.68	− 1.06 ± 1.69	0.10	0.72	0.15 (− 0.65, 0.94)	0.00
Brain-to-body weight ratio	9.78 (8.95, 10.4)	10.5 (9.54, 12.0)	0.09	0.19	0.55 (− 0.28, 1.36)	0.03
Stunting at birth^2^ (*n* [%])	4 (25.0)	7 (46.7)	0.45	–	1.75 (0.65, 4.75)	–
Apgar 1-min score	9.00 (8.25, 9.00)	8.00 (8.00, 9.00)	0.02	0.17	0.58 (− 0.25, 1.39)	0.03
Apgar 5-min score	9.50 (9.00, 10.0)	9.00 (9.00, 9.00)	0.006	0.02	1.05 (0.18, 1.90)	0.24
* Infant anthropometry in infants aged 12 weeks*^1^						
Head circumference *z*-score	− 1.47 (− 2.21, − 0.88)	− 1.07 (− 1.94, − 0.62)	0.32	0.90	0.05 (− 0.74, 0.84)	0.00
Weight *z*-score	− 0.08 (− 0.66, 0.26)	− 0.46 (− 1.47, 0.11)	0.28	0.07	0.82 (− 0.08, 1.69)	0.08
Length *z*-score	− 0.85 ± 0.61	− 1.60 ± 1.02	0.02	0.002	1.38 (0.48, 2.25)	0.22
BMI *z*-score	0.41 ± 0.96	0.11 ± 1.82	0.58	0.43	0.35 (− 0.51, 1.20)	0.00
Brain-to-body weight ratio	7.21 (6.88, 7.69)	7.72 (7.29, 8.12)	0.03	0.17	0.75 (− 0.32, 1.80)	0.04
Weight gain (birth to 12 weeks PP; kg/day)	0.03 ± 0.01	0.03 ± 0.01	0.38	0.17	0.64 (− 0.27, 1.54)	0.04
Stunting at 12 weeks postpartum^2^ (*n* [%])	1 (6.25)	4 (26.7)	0.17	–	4.27 (0.54, 34.0)	–
*Attainment of all 1–3-month GMCD milestones*^3^ *(n [%])*	*n* = 14	*n* = 8				
Expressive language	14 (100)	7 (87.5)	0.36	–	0.88 (0.67, 1.14)	–
Receptive language	11 (78.6)	7 (87.5)	1.00	–	1.11 (0.76, 1.63)	–
Large movement	13 (92.9)	6 (75.0)	0.53	–	0.91 (0.53, 1.24)	–
Fine movement	14 (100)	8 (100)	–	–	–	–
Relating and response behaviour	13 (92.9)	7 (87.5)	1.00	–	0.94 (0.70, 1.27)	–
Play behaviour	12 (85.7)	6 (75.0)		–	0.88 (0.56, 1.38)	–
**Are you able to afford balanced meals?**						
	**Yes**	**No**	***p*** **value (unadjusted)**	***p*** **value (adjusted)**	**Hedge’s** ***g*** **or RR (95% CI)**	***ω***^**2**^
	*n* = 9	*n* = 22				
Gestational age at delivery (weeks)	38.2 ± 1.99	38.7 ± 1.62	0.51	–	–	–
EBF at 12 weeks PP (*n* [%])	8 (88.9)	9 (42.9)	0.04	–	0.51 (0.31, 0.85)	–
* Infant anthropometry and Apgar at birth*^1^						
Head circumference *z*-score	− 1.71 ± 1.14	− 0.87 ± 1.30	0.10	0.13	0.68 (− 0.20, 1.55)	0.05
Weight *z*-score	− 0.83 ± 0.93	− 0.87 ± 1.14	0.94	0.99	0.004 (− 0.85, 0.85)	0.00
Length *z*-score	− 0.98 ± 1.35	− 1.18 ± 1.99	0.79	0.19	0.59 (− 0.30, 1.46)	0.03
BMI *z*-score	− 0.64 ± 1.61	− 0.50 ± 1.83	0.84	0.35	0.42 (− 0.45, 1.29)	0.00
Brain-to-body weight ratio	9.54 (8.97, 10.4)	10.1 (9.38, 11.4)	0.31	0.37	0.40 (− 0.47, 1.25)	0.00
Stunting at birth^2^ (*n* [%])	2 (22.2)	9 (40.9)	0.42	–	1.93 (0.52, 7.21)	–
Apgar 1-min score	9.00 (8.50, 9.00)	8.00 (8.00, 9.00)	0.11	0.11	0.73 (− 0.16, 1.60)	0.05
Apgar 5-min score	10.0 (9.00, 10.0)	9.00 (9.00, 9.00)	0.04	0.06	0.88 (− 0.02, 1.76)	0.10
* Infant anthropometry in infants aged 12 weeks*^1^						
Head circumference *z*-score	− 1.47 (− 2.17, − 0.80)	− 1.34 (− 1.95, − 0.79)	0.71	0.48	0.31 (− 0.55, 1.16)	0.00
Weight *z*-score	− 0.41 (− 0.83, 0.38)	− 0.13 (− 1.12, 0.14)	0.77	0.11	0.75 (− 0.16, 1.64)	0.06
Length *z*-score	− 1.14 (− 1.45, − 0.69)	*− 1.02 (− 1.80, − 0.38)*	0.80	0.21	0.55 (− 0.32, 1.41)	0.02
BMI *z*-score	0.64 ± 1.00	0.12 ± 1.55	0.36	0.18	0.62 (− 0.28, 1.51)	0.04
Brain-to-body weight ratio	7.48 (7.05, 7.68)	7.59 (7.03, 7.88)	0.56	0.41	0.39 (− 0.52, 1.28)	0.00
Weight gain (birth to 12 weeks PP; kg/day)	0.03 ± 0.01	0.03 ± 0.01	0.44	0.08	0.83 (− 0.10, 1.73)	0.08
Stunting at 12 weeks postpartum^2^ (*n* [%])	1 (11.0)	4 (18.2)	1.00	–	1.64 (0.21, 12.7)	–
* Attainment of all 1–3-month GMCD milestones*^3^ *(n[%])*	*n* = 7	*n* = 15				
Expressive language	7 (100)	14 (93.3)	1.00	–	0.93 (0.82, 1.07)	–
Receptive language	6 (85.7)	12 (80.0)	1.00	–	0.93 (0.63, 1.39)	–
Large movement	7 (100)	12 (80.0)	0.52	–	0.80 (0.62, 1.03)	–
Fine movement	7 (100)	15 (100)	–	–	–	–
Relating and response behaviour	7 (100)	13 (86.7)	1.00	–	0.87 (0.71, 1.06)	–
Play behaviour	7 (100)	11 (73.3)	0.26	–	0.73 (0.54, 1.00)	–

Data from mother-infant dyads that attended follow-up are presented mean ± SD (ANOVA to test equality of means for normal data with equal variance) or median (IQR; Kruskal-Wallis/Wilcoxon test to test equality of medians for non-parametric data or Welch’s test to test equality of means for normal data with unequal variance) with *p* values from one-way analysis of variance and adjusted multiple variable regression models. Hedge’s *g* and RR are reported as effect size estimates for continuous and categorical infant outcome data, respectively. Omega square (*ω*^2^) denotes variance accounted for by the model. Differences in proportion of infants exclusively breastfed (EBF) at follow-up were assessed by Fisher’s exact test (2-tail). Infant anthropometry was adjusted for maternal age, maternal weight and gestational age. BBR, Apgar score (1 and 5 min) and weight gain (birth to 12 weeks PP; kg/day) were adjusted for maternal age, maternal weight, gestational age and infant sex

*RR* relative risk, *PP* postpartum

^1^All infant anthropometric measures are standardised according to WHO child growth standards [28]

^2^Stunting is determined by <− 2 SD length-for-age standardised *z*-score according to WHO child growth standards [28]

^3^For fine movement milestones at 1–3 months, all infants in both groups met all milestones so no comparisons were made. There was only 1 HUU infant who was 3–5 months at follow-up, so no comparisons for GMCD 3–5-month milestones were made between the two groups

#### Influence of food insecurity on infant Apgar scores and growth outcomes at birth

Maternal reports of food insecurity did not appear to influence infant gestational age or infant anthropometry at birth (Table 4). Apgar score at 5 min was slightly lower for infants whose mothers reported worrying about (9.00 [9.00, 9.00] vs. 10.0 [9.00, 10.0], *p* = 0.01) or experiencing (9.00 [9.00, 9.00] vs. 9.50 [9.00, 10.0], *p* = 0.02) food runout compared to those who reported not worrying about or experiencing food runout (Table 4).

#### Influence of food insecurity on infant growth outcomes at 12 weeks

Infant length at 12 weeks was lower among infants whose mothers reported worrying about (− 1.52 ± 0.99 vs. − 0.84 ± 0.64, *p* = 0.01) and experiencing (− 1.60 ± 1.02 vs. − 0.85 ± 0.61, *p* = 0.002) food runout in comparison to those who did not (Table 4). There were no other effects of food security on infant anthropometry at 12 weeks (Table 4).

#### HEU may increase vulnerability to effects of food insecurity on the risk of stunting at birth

Among infants whose mothers reported worrying about food runout, infants who were HEU had an increased risk of stunting at birth compared to infants who were HUU (RR = 4.90 [0.76, 31.5], ARD = 0.56 [0.17, 0.94], *p* = 0.0498; [Supplementary fig 4A]). Maternal HIV status did not influence the relationship between maternal reports of food insecurity and any other infant outcomes at birth or 12 weeks postpartum.

#### Influence of food insecurity on neurodevelopmental outcomes in 12-week-old infants

The probability of attaining all 1–3-month GMCD expressive and receptive language, large movement, play activities and relating and response behaviour milestones did not associate with maternal reports of household food insecurity (Table 4; Supplementary fig S5). No comparisons were made for fine movement outcomes between infants exposed to food-insecure conditions compare to those who were not, as all infants who were 1–3 months of age at follow-up met all age-appropriate fine movement milestones. At 3–5 months, there were no differences in the proportion of infants who attained fine movement and relating and response behaviour milestones based on maternal reports of food security (Table 4; Supplementary fig S5). No comparisons were made for expressive and receptive language, large movement or play activities, as all infants 3–5 months of age at follow-up had met these milestones. It was not possible to further stratify these comparisons based on maternal HIV status due to the small sample size of the pilot.

## Discussion

As access to ART increases worldwide and the number of infants born each year who are HIV-exposed but uninfected also rises, there is a growing need to understand how, and to what extent, exposure to HIV and ART in utero and during breastfeeding influences infant health trajectories. In this small pilot study, we first show we can recruit a cohort of women living with and without HIV to investigate the effects of HEU on growth and immune- and neurodevelopment in infants in early life, although there remain barriers to long-term follow-up. Next, our hypothesis-generating analyses revealed that infants who are HEU may have reduced head circumference and elevated CCR2 expression by CD14^+^ monocytes within 4 days of birth compared to infants who are HUU. Last, our exploratory analyses also suggest that food insecurities, and the likely ensuing poor maternal nutritional status, may adversely affect the growth and neurodevelopment of infants in the first 4 months of life, and at least for some measures, the effects of a suboptimal early life nutritional environment may be most detrimental for infants who are HEU. While these exploratory analyses are underpowered to make conclusive statements, findings will inform our research questions and analyses in our full-scale, observational study aimed at better understanding these exposure-outcome relationships.

Our finding that infants who are HEU may have reduced head circumference at birth compared to infants who are HUU is in agreement with other studies, which found that HEU associates with lower weight, length, BMI [41, 42]^,^ and head circumference [43] at birth compared to infants who are HUU. Small head circumference at birth has been shown to associate with poorer performance on neurodevelopmental assessments in school-aged children, including on cognitive tasks measuring memory and visuo-spatial ability [44], early adiposity rebound and increased risk for adult obesity [45, 46], cardiovascular disease mortality [47] and mental health disorders such as schizophrenia [48]. It is not known whether, in the context of maternal HIV infection, small head circumference at birth is linked to persistent deficits in neurodevelopment and/or, in the longer term, later life brain and metabolic compromise. However, the effect of HEU on neurodevelopment and growth outcomes will be further investigated in our full-scale study over the first 2 years.

We also observed elevated CCR2 expression on classical monocytes at birth and increased levels of CCR2 expression (MFI) on intermediate monocytes at birth and 12 weeks in infants who were HEU. Increased CCR2 expression may result in increased recruitment of monocyte populations across the blood-brain barrier, which may have consequences for neurodevelopment, as the expression of CCR2 by CD14^+^ monocytes has been shown to associate with HIV-1-induced neuropsychological impairment and neuroinflammation in adults [21, 49]. The expression and release of a CCR2 ligand, monocyte chemoattractant protein-1 (MCP-1), from astrocytes in the brain is increased by HIV-1 infection [50]. MCP-1 levels have been shown to positively correlate with the severity of HIV-induced neuropsychological impairment in adults [51]. Importantly, whether or not elevated MCP-1 levels or CCR2 expression by monocyte subsets have consequences for children perinatally exposed to HIV remains to be determined and will be examined further in our scaled-up cohort.

When exploring relationships between food security and infant outcomes, we found that household food insecurity may associate with reduced infant length at 12 weeks postpartum, and infants who also experience HEU may be at an increased risk of stunting at birth compared to infants who are HUU and whose mothers also experience food-insecure conditions. Stunting is the most common manifestation of infant undernutrition globally [52]. Our findings may suggest that infants who are HEU are distinctly vulnerable to the programming effects of suboptimal nutrition in utero and postnatally, and future studies should further investigate the mechanisms that may underly this relationship.

Differences in maternal nutrient intakes between mothers living with and without HIV were minimal, and mothers were at risk for inadequate macronutrient intakes irrespective of food insecurity reports. We also found maternal reports of experiencing food insecurity associated with lower vitamin B_12_, and a large proportion of mothers, irrespective of food insecurity circumstances, were at risk for inadequate intakes. Inadequate maternal vitamin B_12_ intakes have shown to cause secondary deficiencies in breastfed infants in the first 6 months of life, leading to delayed growth and neurodevelopment [53]. Food insecurity is also a known barrier to exclusive breastfeeding [54], and mothers experiencing food insecurity may be more likely to return to work soon after birth [55] or may have challenges maintaining milk supply due to inadequate nutrition [56]. In agreement with this, we found that mothers who never experienced food runout or were always able to afford balanced meals were more likely to be exclusively breastfeeding at follow-up. Given the high rates of reported food insecurity among the pilot study cohort and the potential influence of food and nutrition insecurity on maternal nutrient intakes, we will be including these important variables in our full-scale study, which will allow us to assess relationships between food and nutrition insecurity, maternal HIV infection, and infant feeding practices and development over a 24-month period, encompassing the recommended EBF (6 months) and mixed feeding (24 months) periods [57].

### Limitations to the study

The translatability of the analyses presented are restricted by the pilot’s small sample size, limiting statistical power, and because model assumptions were affected due to non-normal data distribution and sample size. These exploratory analyses were intended to inform the development and refinement of our research questions and study protocol for a full-scale study investigating relationships between the early life nutritional environment, HEU and infant development. There was high attrition among both populations, particularly within the HUU group, which may have led to sample bias at follow-up. Individuals from communities in this area are highly mobile, and postpartum women, in particular, may be especially mobile [58]. It is possible that women recruited into the cohort had relocated to, or been within, our catchment area for antenatal care and, following delivery, returned to their homes in more rural locations for familial support, as has been discussed in other studies [58, 59]. Although multiple attempts were made to contact each mother to encourage her to return, this presented challenges for study retention. Missing data, including immune measures at birth for one infant who was HUU and 12 weeks for four infants who were HEU, as well as a GMCD assessment for one infant who was HUU, may have further biased these results and limit the translatability of our findings. Our full-scale study will handle missing data according to recommended guidelines for longitudinal, observational cohort studies [60]. Despite these limitations, our study was able to capture information on infant growth, immune function and neurodevelopment at two time points within the same infant population. We are currently conducting a larger prospective pregnancy and birth cohort study at Kalafong Hospital to further investigate relationships that have emerged in these exploratory pilot study analyses. In an aim to improve participant retention in the full-scale study, Ward-based Primary Health Care Outreach teams have been employed to trace and contact women who miss a follow-up appointment. To our knowledge, this prospective cohort study is among the few to concomitantly interrogate growth and neurodevelopmental outcomes, immune function, and maternal nutrition and food security among a population of infants who are HEU in comparison to infants who are HUU.

## Conclusions

Study participant retention was challenging in this pilot; however, the study helped to identify barriers to recruitment and retention that were used to inform a revised full-scale study protocol. Exploratory data analyses revealed possible relationships between exposure to maternal HIV infection in the womb, household food insecurity and infant outcomes at birth and 12 weeks postpartum. We are now investigating these relationships in a full-scale, longitudinal observational study.

## Supplementary Information

**Additional file 1: **Supplementary **Figure S1**. Sequential gating approach for the measurement of CCR2 expression by CD14+ monocytes. The sequential gating approached used was as follows: First, the viable (7-AAD negative; region ‘Viable”) cells were identified using a 7-AAD vs SS Log density plot. A “Viable” region was created around the 7-AAD negative cells. Gated on the “Viable”cells, a SSLog vs FS plot was used to capture intact cells in the “E” region. CD14+ monocytes were identified (“CD14+” region) using a CD14 vs SS Log density plot that were gated on viable, intact cells (“E” region). CD14+ monocytes that express CCR2 were quantified using a CD192 (CCR2) vs SS Log plot. The proportion of CD14+/CCR2+ cells were captured in the “CD14+ CCR2+” region. The gating strategy followed to quantify CCR2 expression by CD16+ neutrophils was similar to what was described for CD14+ monocytes, but instead of identifying CD14+ monocytes, CD16+ neutrophils were identified (“CD16+” region) using a CD16 vs SS Log density plot that were gated on viable, intact cells (“E” region). CD16+ neutrophils that express CCR2 were quantified using a CD192 (CCR2) vs SS Log plot. The proportion of CD14+/CCR2+ cells was captured in the “CD16+ CCR2+” region.

**Additional file 2: **Supplementary **Figure S2.** Sequential gating approach for the measurement of CCR2 expression by monocyte subpopulations. Doublets and debris were removed (Region ‘K’) using a FS Area vs FS Height density plot. A 7-AAD vs SS Log density plot, gated on ‘K’ was used to exclude all non-viable cells. Viable cells were captured in region ‘Viable’. Viable CD14+ monocytes were identified (Region ‘CD14+ Monocytes’) using a CD14 APC vs SS Log density plot. Monocyte sub-populations were identified using a CD16 FITC vs CD14 PE density plot gated on viable, CD14+ monocytes. Four monocyte sub-populations were identified: CD14+/CD16-; CD14++/CD16-; CD14+/CD16+; and CD14++/CD16+. The percentage CCR2+ monocytes present in each of the respective monocyte sub-populations were identified using CD195 (CCR2) PE vs SS Log two-parameter plots gated on the respective sub-populations. The overlay plots within the black bordered square indicates the strategy used to determine CCR2 expression of the different monocyte subsets. The negative/positive staining boundaries were determined based on the negative expression of CCR2 by CD16++/CD14- neutrophils (indicated in red in the overlay plots). The CCR2+ populations are indicated in blue.

**Additional file 3: **Supplementary **Figure S3**. Maternal intake of estimated average requirements for macronutrients, vitamins and minerals for mothers who report on household food security circumstances. Maternal reports of food insecurity did not associate with intake levels of macronutrients or minerals. Maternal reports of experiencing food runout or inability to afford balanced meals associated with lower intake of vitamin B12 (p=0.01; p=0.04). Many women, irrespective of food security reports, are at risk of inadequate macronutrient, vitamin and mineral intakes. Percent intake of EARs for 36 nutrients were calculated for lactating women 14-18, 19-30 or 31-50 years of age [37]. Calculations for EAR for total protein considered maternal weight at time of dietary recall. Data are % intake of EAR reported in maternal dietary recall for macronutrients, *p<0.05 [ANOVA for normal distribution/equal variance; Kruskal-Wallis/Wilcoxon test for nonparametric data; or Welch’s test for normal data/unequal variance]). CHO = carbohydrates.

**Additional file 4: **Supplementary **Figure S4**. Cooccurrence of maternal HIV and food insecurity may increase risk of stunting at birth. Amongst infants whose mothers report worrying about food runout, risk of stunting at birth is greater for HEU compared to HUU infants (*e*; RR=4.90 [0.76, 31.5], ARD=0.56 [0.17, 0.94], p=0.0498). The red line represents the proportion of infants who had stunting at birth or 12 weeks PP. Mosaic plots are proportion (%) of HUU or HEU infants who have stunting (<-2 SD length-for-age standardised according to WHO child growth standards [28]) at birth and 12 weeks old. HUU = HIV-unexposed, uninfected infant; HEU = HIV-exposed, uninfected infant. RR = Relative risk. ARD = Absolute risk difference.

**Additional file 5: **Supplementary **Figure S5**. Food insecurity may associate with low attainment of GMCD milestones for HUU and HEU infants. Infants whose mothers reported household food insecurity did not attain 1-3 month GMCD milestones (A, C, E) for receptive language, large movement, relating and response behaviour or play activities, or 3-5 month GMCD milestones (B, D, F) for fine movement or relating and response behaviour in the same proportion as the international standardization sample. Maternal reports of food insecurity did not associate with risk of not attaining all 1-3 month or 3-5 month GMCD milestones (A-F, [p>0.05], Fisher’s exact 2-Tail). Data are proportion (%) of infants who attained all age-appropriate GMCD milestones. The horizontal dotted line represents the GMCD standardised international sample proportion (85%) of infants who attained all milestones in that age category, when they were in that age range. The numbers underneath the bars represent the number of infants attaining all milestones for each milestone. GMCD = Guide for monitoring child development; HUU = HIV-unexposed, uninfected infant; HEU = HIVexposed, uninfected infant.

**Additional file 6: Supplementary Table S1.** Flow cytometry reagent list (including lasers and detectors used). **Supplementary Table S2.** Flow cytometry compensation matrix. **Supplementary Table S3.** Maternal nutrient intakes from one 24-hour dietary recall for mothers with and without HIV who attended follow up. **Supplementary Table S4.** Maternal nutrient intake from one 24-hour dietary recall for mothers who report experiencing food insecurity compared to those who do not experience food insecurity.

## Data Availability

The datasets analysed during the current study are available from the corresponding author on reasonable request.
