# Supplementary material for: Does in utero HIV exposure and the early nutritional environment influence infant development and immune outcomes? Findings from a pilot study in Pretoria, South Africa
Source: Pilot Feasibility Stud. 2020 Dec 11;6:192. doi: 10.1186/s40814-020-00725-8 (PMC7730756; doi:10.1186/s40814-020-00725-8)
Supplement: Supplementary file 1 — Additional file 1: Supplementary Figure S1. Sequential gating approach for the measurement of CCR2 expression by CD14+ monocytes. The sequential gating approached used was as follows: First, the viable (7-AAD negative; region ‘Viable”) cells were identified using a 7-AAD vs SS Log density plot. A “Viable” region was created around the 7-AAD negative cells. Gated on the “Viable”cells, a SSLog vs FS plot was used to capture intact cells in the “E” region. CD14+ monocytes were identified (“CD14+” region) using a CD14 vs SS Log density plot that were gated on viable, intact cells (“E” region). CD14+ monocytes that express CCR2 were quantified using a CD192 (CCR2) vs SS Log plot. The proportion of CD14+/CCR2+ cells were captured in the “CD14+ CCR2+” region. The gating strategy followed to quantify CCR2 expression by CD16+ neutrophils was similar to what was described for CD14+ monocytes, but instead of identifying CD14+ monocytes, CD16+ neutrophils were identified (“CD16+” region) using a CD16 vs SS Log density plot that were gated on viable, intact cells (“E” region). CD16+ neutrophils that express CCR2 were quantified using a CD192 (CCR2) vs SS Log plot. The proportion of CD14+/CCR2+ cells was captured in the “CD16+ CCR2+” region. [file 40814_2020_725_MOESM1_ESM.pdf]

Analysis approach and gating strategy: CCR2 expression on CD14+ monocytes

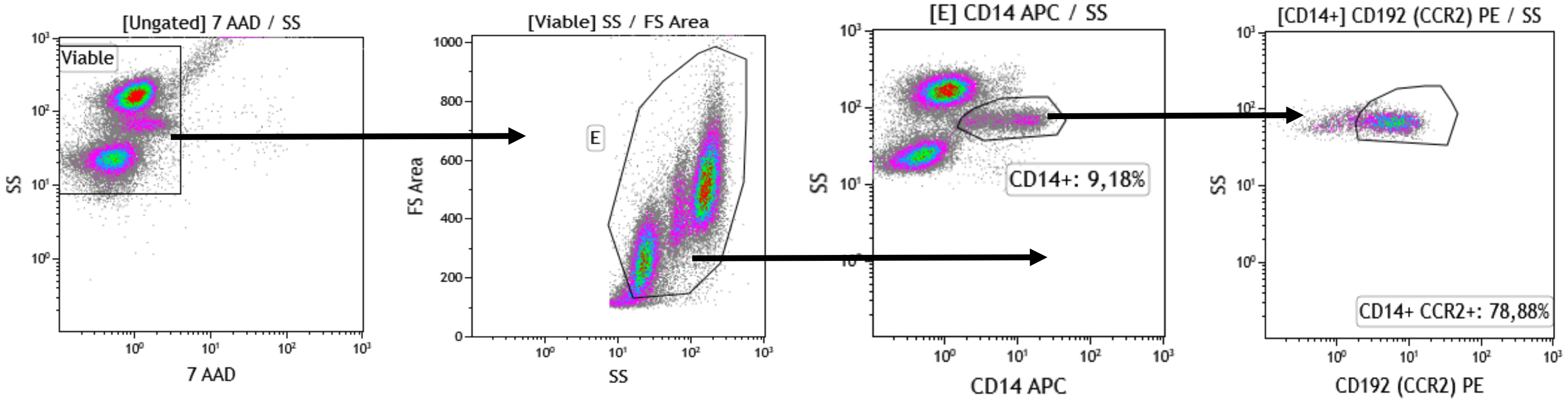

Supplementary figure S1. Sequential gating approach for the measurement of CCR2 expression by CD14+ monocytes. The sequential gating approached used was as follows: First, the viable (7-AAD negative; region 'Viable'') cells were identified using a 7-AAD vs SS Log density plot. A "Viable" region was created around the 7-AAD negative cells. Gated on the "Viable"cells, a SSLog vs FS plot was used to capture intact cells in the "E" region. CD14+ monocytes were identified ("CD14+" region) using a CD14 vs SS Log density plot that were gated on viable, intact cells ("E" region). CD14+ monocytes that express CCR2 were quantified using a CD192 (CCR2) vs SS Log plot. The proportion of CD14+/CCR2+ cells were captured in the "CD14+ CCR2+" region. The gating strategy followed to quantify CCR2 expression by CD16+ neutrophils was similar to what was described for CD14+ monocytes, but instead of identifying CD14+ monocytes, CD16+ neutrophils were identified ("CD16+" region) using a CD16 vs SS Log density plot that were gated on viable, intact cells ("E" region). CD16+ neutrophils that express CCR2 were quantified using a CD192 (CCR2) vs SS Log plot. The proportion of CD14+/CCR2+ cells was captured in the "CD16+ CCR2+" region.
