# Supplementary material for: Does in utero HIV exposure and the early nutritional environment influence infant development and immune outcomes? Findings from a pilot study in Pretoria, South Africa
Source: Pilot Feasibility Stud. 2020 Dec 11;6:192. doi: 10.1186/s40814-020-00725-8 (PMC7730756; doi:10.1186/s40814-020-00725-8)
Supplement: Supplementary file 2 — Additional file 2: Supplementary Figure S2. Sequential gating approach for the measurement of CCR2 expression by monocyte subpopulations. Doublets and debris were removed (Region ‘K’) using a FS Area vs FS Height density plot. A 7-AAD vs SS Log density plot, gated on ‘K’ was used to exclude all non-viable cells. Viable cells were captured in region ‘Viable’. Viable CD14+ monocytes were identified (Region ‘CD14+ Monocytes’) using a CD14 APC vs SS Log density plot. Monocyte sub-populations were identified using a CD16 FITC vs CD14 PE density plot gated on viable, CD14+ monocytes. Four monocyte sub-populations were identified: CD14+/CD16-; CD14++/CD16-; CD14+/CD16+; and CD14++/CD16+. The percentage CCR2+ monocytes present in each of the respective monocyte sub-populations were identified using CD195 (CCR2) PE vs SS Log two-parameter plots gated on the respective sub-populations. The overlay plots within the black bordered square indicates the strategy used to determine CCR2 expression of the different monocyte subsets. The negative/positive staining boundaries were determined based on the negative expression of CCR2 by CD16++/CD14- neutrophils (indicated in red in the overlay plots). The CCR2+ populations are indicated in blue. [file 40814_2020_725_MOESM2_ESM.pdf]

Analysis approach and gating strategy: CCR2 expression by monocyte sub-populations

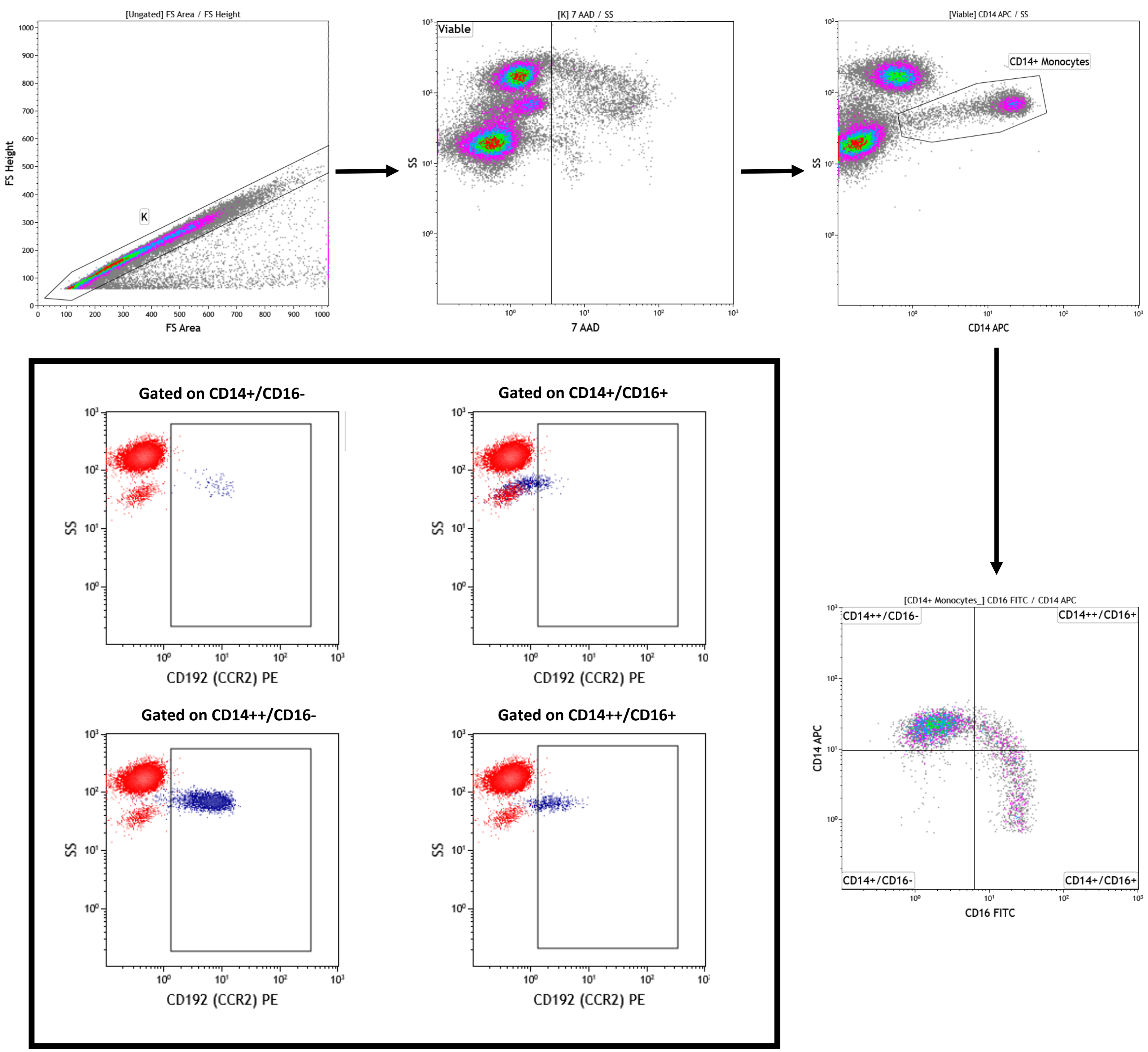

Supplementary figure S2. Sequential gating approach for the measurement of CCR2 expression by monocyte sub-populations. Doublets and debris were removed (Region 'K') using a FS Area vs FS Height density plot. A 7-AAD vs SS Log density plot, gated on 'K' was used to exclude all non-viable cells. Viable cells were captured in region 'Viable'. Viable CD14+ monocytes were identified (Region 'CD14+ Monocytes') using a CD14 APC vs SS Log density plot. Monocyte sub-populations were identified using a CD16 FITC vs CD14 PE density plot gated on viable, CD14+ monocytes. Four monocyte sub-populations were identified: CD14+/CD16-; CD14++/CD16-; CD14+/CD16+; and CD14++/CD16+. The percentage CCR2^{+} monocytes present in each of the respective monocyte sub-populations were identified using CD195 (CCR2) PE vs SS Log two-parameter plots gated on the respective sub-populations. The overlay plots within the black bordered square indicates the strategy used to determine CCR2 expression of the different monocyte subsets. The negative/positive staining boundaries were determined based on the negative expression of CCR2 by CD16^{++}/CD14^{-} neutrophils (indicated in red in the overlay plots). The CCR2^{+} populations are indicated in blue.
