# Supplementary material for: Does in utero HIV exposure and the early nutritional environment influence infant development and immune outcomes? Findings from a pilot study in Pretoria, South Africa
Source: Pilot Feasibility Stud. 2020 Dec 11;6:192. doi: 10.1186/s40814-020-00725-8 (PMC7730756; doi:10.1186/s40814-020-00725-8)
Supplement: Supplementary file 3 — Additional file 3: Supplementary Figure S3. Maternal intake of estimated average requirements for macronutrients, vitamins and minerals for mothers who report on household food security circumstances. Maternal reports of food insecurity did not associate with intake levels of macronutrients or minerals. Maternal reports of experiencing food runout or inability to afford balanced meals associated with lower intake of vitamin B12 (p=0.01; p=0.04). Many women, irrespective of food security reports, are at risk of inadequate macronutrient, vitamin and mineral intakes. Percent intake of EARs for 36 nutrients were calculated for lactating women 14-18, 19-30 or 31-50 years of age [37]. Calculations for EAR for total protein considered maternal weight at time of dietary recall. Data are % intake of EAR reported in maternal dietary recall for macronutrients, *p<0.05 [ANOVA for normal distribution/equal variance; Kruskal-Wallis/Wilcoxon test for nonparametric data; or Welch’s test for normal data/unequal variance]). CHO = carbohydrates. [file 40814_2020_725_MOESM3_ESM.pdf]

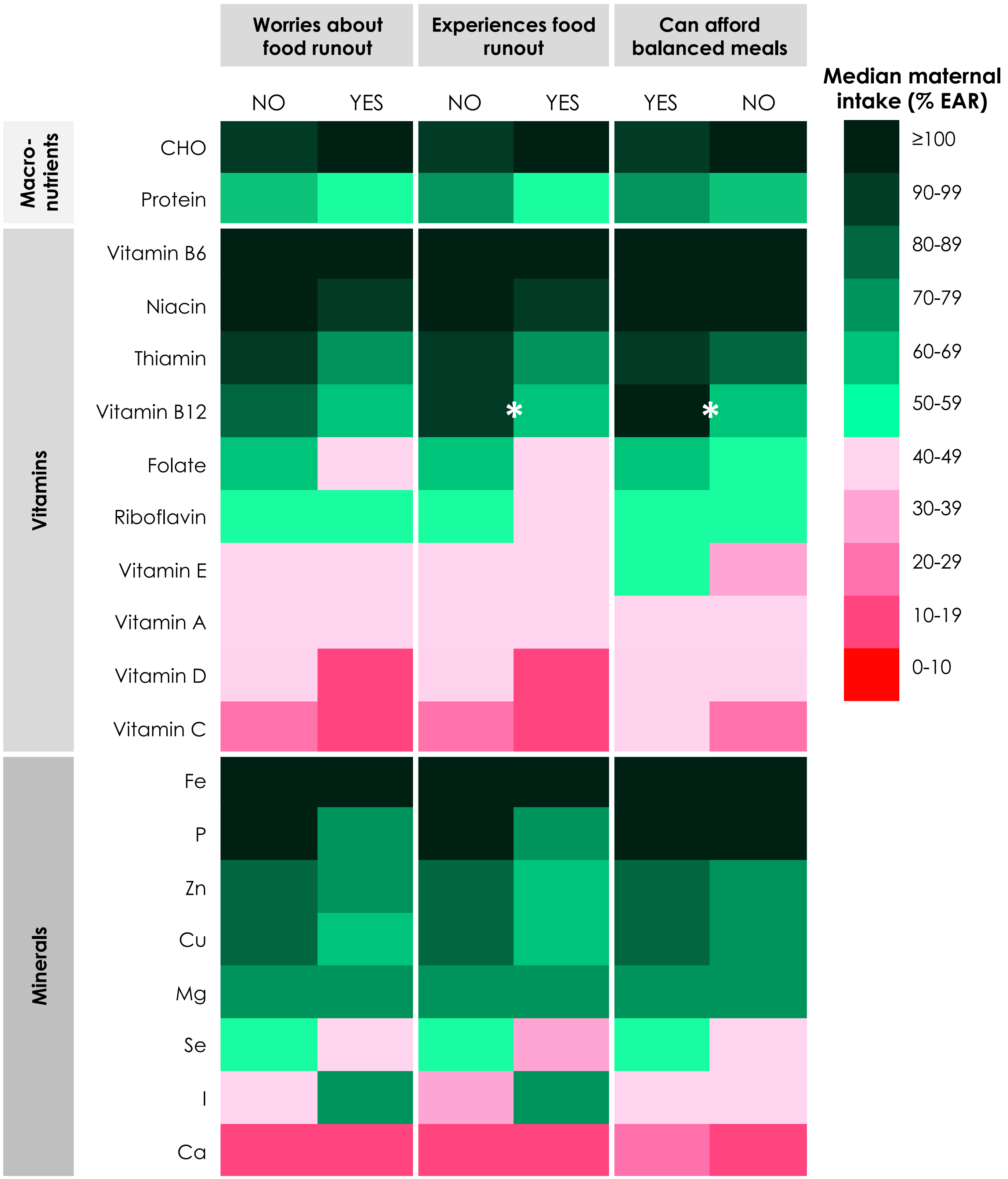

Supplementary figure S3. Maternal intake of estimated average requirements for macronutrients, vitamins and minerals for mothers who report on household food security circumstances. Maternal reports of food insecurity did not associate with intake levels of macronutrients or minerals. Maternal reports of experiencing food runout or inability to afford balanced meals associated with lower intake of vitamin B12 ($p=0.01$; $p=0.04$). Many women, irrespective of food security reports, are at risk of inadequate macronutrient, vitamin and mineral intakes. Percent intake of EARs for 36 nutrients were calculated for lactating women 14-18, 19-30 or 31-50 years of age [37]. Calculations for EAR for total protein considered maternal weight at time of dietary recall. Data are % intake of EAR reported in maternal dietary recall for macronutrients, *$p<0.05$ [ANOVA for normal distribution/equal variance; Kruskal-Wallis/Wilcoxon test for non-parametric data; or Welch's test for normal data/unequal variance]). CHO = carbohydrates.
