# Supplementary material for: Does in utero HIV exposure and the early nutritional environment influence infant development and immune outcomes? Findings from a pilot study in Pretoria, South Africa
Source: Pilot Feasibility Stud. 2020 Dec 11;6:192. doi: 10.1186/s40814-020-00725-8 (PMC7730756; doi:10.1186/s40814-020-00725-8)
Supplement: Supplementary file 4 — Additional file 4: Supplementary Figure S4. Cooccurrence of maternal HIV and food insecurity may increase risk of stunting at birth. Amongst infants whose mothers report worrying about food runout, risk of stunting at birth is greater for HEU compared to HUU infants (e; RR=4.90 [0.76, 31.5], ARD=0.56 [0.17, 0.94], p=0.0498). The red line represents the proportion of infants who had stunting at birth or 12 weeks PP. Mosaic plots are proportion (%) of HUU or HEU infants who have stunting (<-2 SD length-for-age standardised according to WHO child growth standards [28]) at birth and 12 weeks old. HUU = HIV-unexposed, uninfected infant; HEU = HIV-exposed, uninfected infant. RR = Relative risk. ARD = Absolute risk difference. [file 40814_2020_725_MOESM4_ESM.pdf]

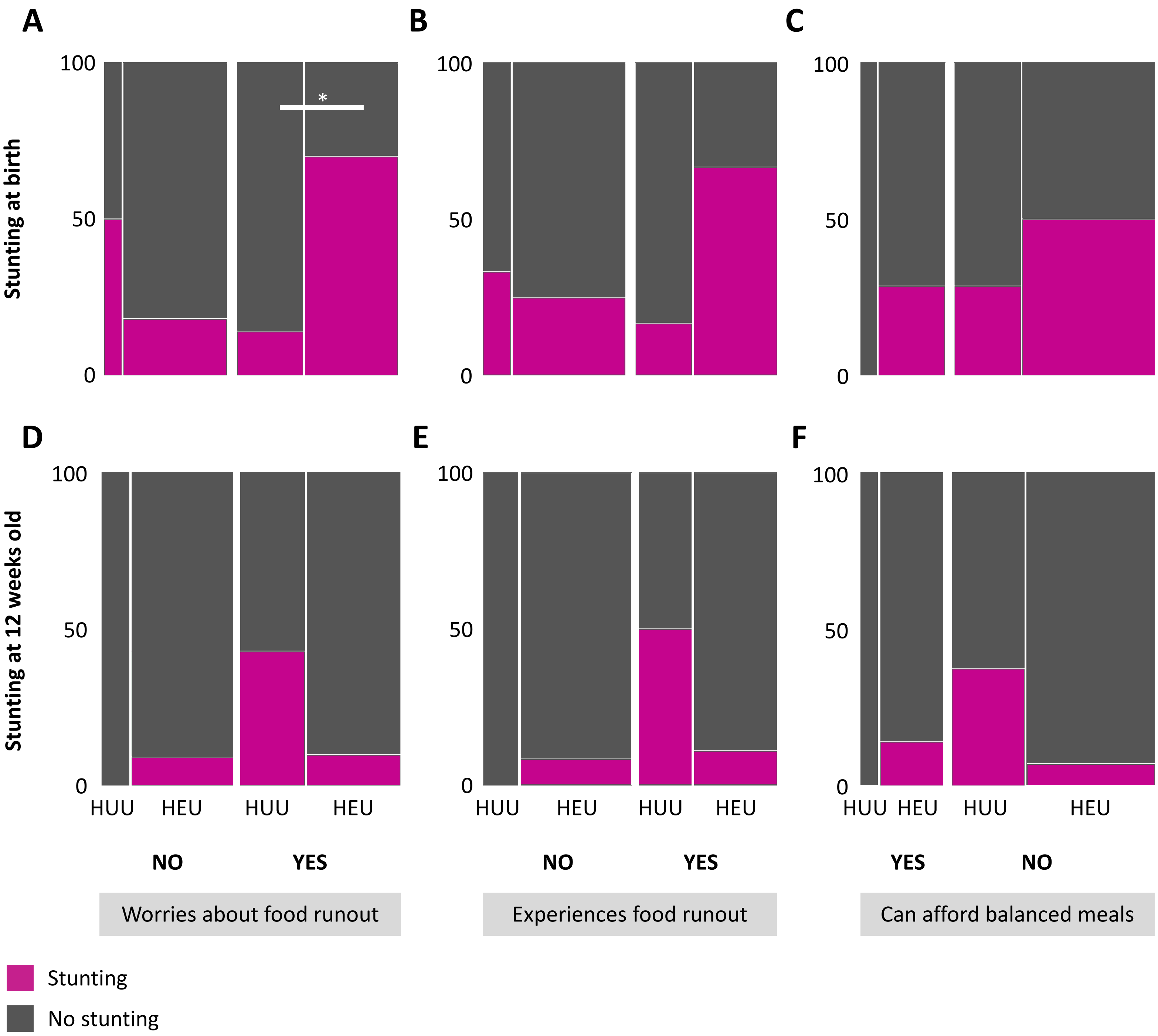

Supplementary figure S4. Cooccurrence of maternal HIV and food insecurity may increase risk of stunting at birth. Amongst infants whose mothers report worrying about food runout, risk of stunting at birth is greater for HEU compared to HUU infants (*e*; RR=4.90 [0.76, 31.5], ARD=0.56 [0.17, 0.94], p=0.0498). The red line represents the proportion of infants who had stunting at birth or 12 weeks PP. Mosaic plots are proportion (%) of HUU or HEU infants who have stunting (<-2 SD length-for-age standardised according to WHO child growth standards [28]) at birth and 12 weeks old. HUU = HIV-unexposed, uninfected infant; HEU = HIV-exposed, uninfected infant. RR = Relative risk. ARD = Absolute risk difference.
