# Supplementary material for: Does in utero HIV exposure and the early nutritional environment influence infant development and immune outcomes? Findings from a pilot study in Pretoria, South Africa
Source: Pilot Feasibility Stud. 2020 Dec 11;6:192. doi: 10.1186/s40814-020-00725-8 (PMC7730756; doi:10.1186/s40814-020-00725-8)
Supplement: Supplementary file 5 — Additional file 5: Supplementary Figure S5. Food insecurity may associate with low attainment of GMCD milestones for HUU and HEU infants. Infants whose mothers reported household food insecurity did not attain 1-3 month GMCD milestones (A, C, E) for receptive language, large movement, relating and response behaviour or play activities, or 3-5 month GMCD milestones (B, D, F) for fine movement or relating and response behaviour in the same proportion as the international standardization sample. Maternal reports of food insecurity did not associate with risk of not attaining all 1-3 month or 3-5 month GMCD milestones (A-F, [p>0.05], Fisher’s exact 2-Tail). Data are proportion (%) of infants who attained all age-appropriate GMCD milestones. The horizontal dotted line represents the GMCD standardised international sample proportion (85%) of infants who attained all milestones in that age category, when they were in that age range. The numbers underneath the bars represent the number of infants attaining all milestones for each milestone. GMCD = Guide for monitoring child development; HUU = HIV-unexposed, uninfected infant; HEU = HIVexposed, uninfected infant. [file 40814_2020_725_MOESM5_ESM.pdf]

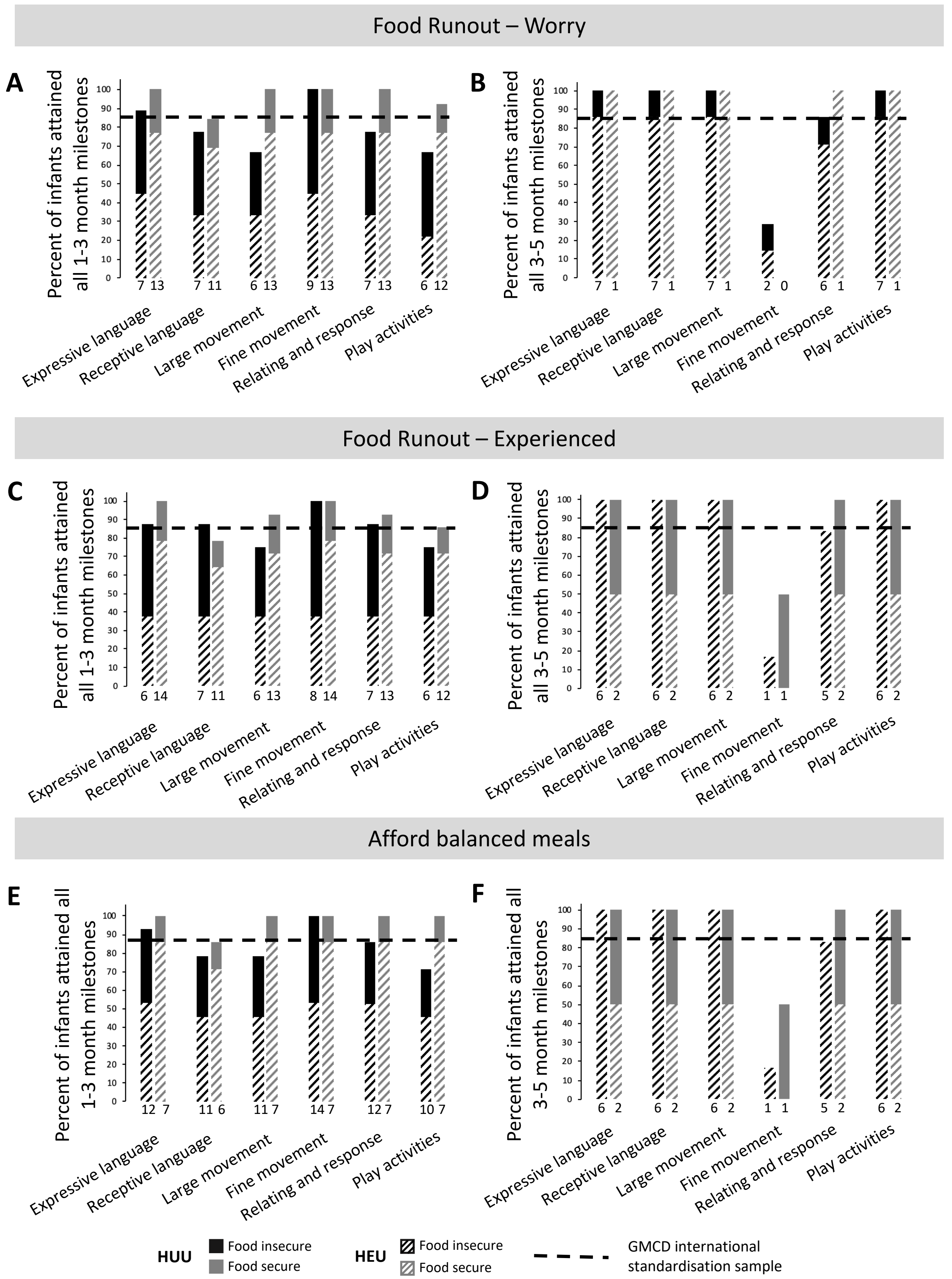

Supplementary figure S5. Food insecurity may associate with low attainment of GMCD milestones for HUU and HEU infants. Infants whose mothers reported household food insecurity did not attain 1-3 month GMCD milestones (A, C, E) for receptive language, large movement, relating and response behaviour or play activities, or 3-5 month GMCD milestones (B, D, F) for fine movement or relating and response behaviour in the same proportion as the international standardization sample. Maternal reports of food insecurity did not associate with risk of not attaining all 1-3 month or 3-5 month GMCD milestones (A-F, [p>0.05], Fisher's exact 2-Tail). Data are proportion (%) of infants who attained all age-appropriate GMCD milestones. The horizontal dotted line represents the GMCD standardised international sample proportion (85%) of infants who attained all milestones in that age category, when they were in that age range. The numbers underneath the bars represent the number of infants attaining all milestones for each milestone. GMCD = Guide for monitoring child development; HUU = HIV-unexposed, uninfected infant; HEU = HIV-exposed, uninfected infant.
