# Supplementary material for: Does in utero HIV exposure and the early nutritional environment influence infant development and immune outcomes? Findings from a pilot study in Pretoria, South Africa
Source: Pilot Feasibility Stud. 2020 Dec 11;6:192. doi: 10.1186/s40814-020-00725-8 (PMC7730756; doi:10.1186/s40814-020-00725-8)
Supplement: Supplementary file 6 — Additional file 6: Supplementary Table S1. Flow cytometry reagent list (including lasers and detectors used). Supplementary Table S2. Flow cytometry compensation matrix. Supplementary Table S3. Maternal nutrient intakes from one 24-hour dietary recall for mothers with and without HIV who attended follow up. Supplementary Table S4. Maternal nutrient intake from one 24-hour dietary recall for mothers who report experiencing food insecurity compared to those who do not experience food insecurity. [file 40814_2020_725_MOESM6_ESM.pdf]

Supplementary table S1. Flow cytometry reagent list (including lasers and detectors used).

Monoclonal antibody	Clone	Supplier	Excitation (laser)	Fluorescent Channel	Emission (Detector
7-AAD	N/A	Beckman Coulter, Miami, FL, USA	488 nm (blue)	FL4	695/30
CD16-FITC	3G8	BioLegend®, San Diego, CA, USA	488 nm (blue)	FL1	525/40
CD14-APC	63D3	BioLegend®, San Diego, CA, USA	635 nm (red)	FL6	660/20
CD192 (CCR2)-PE	K036C2	BioLegend®, San Diego, CA, USA	488 nm (blue)	FL2	575/30

N/A: Not applicable

Supplementary table S2. Flow cytometry compensation matrix.

% Spillover				
	**FL1**	**FL2**	**FL4**	**FL6**
**FL1**	-	2.40	0.00	0.00
**FL2**	5.00	-	0.00	0.00
**FL4**	0.00	59.98	-	0.00
**FL6**	0.00	0.00	0.00	-

Supplementary table S3. Maternal nutrient intakes from one 24-hour dietary recall for mothers with and without HIV who attended follow up.

	**HIV-uninfected** (n=10)	**HIV-infected** (n=21)	**p value**
**Macronutrients – Absolute intakes**			
Energy (kJ)	7339 (3862-7984)	5204 (4024-6590)	NS
Nitrogen (g)	4.15 (2.26-6.29)	6.67 (4.87-8.20)	0.02
Total protein (g)	46.6 (23.7-57.6)	44.0 (33.2-61.6)	NS
Plant protein (g)	26.0 (13.2-40.5)	18.9 (12.5-25.4)	NS
Animal protein (g)	13.8 (3.3-33.5)	27.5 (18.6-35.0)	NS
Total fat (g)	30.6 (22.8-81.4)	33.9 (22.2-53.8)	NS
Carbohydrate, avail. (g)	201 (140 - 282)	160 (136-209)	NS
Starch (g)	0.00 (0-4.55)	5.50 (0.1-9.3)	0.04
Glucose (g)	0.20 (0-5.8)	3.70 (1.8-4.55)	NS
Fructose (g)	0.20 (0-7.35)	6.10 (0.65-6.8)	NS
Galactose (g)	0.00 (0-0)	0.00 (0-0)	-
Sucrose (g)	10.2 (0-33.3)	15.1 (11.8-22.1)	NS
Maltose (g)	0.00 (0-0)	0.00 (0-0)	-
Lactose (g)	0.00 (0-3.38)	0.00 (0-0.2)	NS
Total sugars (g)	23.1 (6.83-33.8)	29.0 (14.5-38.4)	NS
Added sugar (g)	6.35 (0.53-67.0)	13.4 (10.6-26.05)	NS
Non-starch polysaccharides (g)	0.90 (0-10.5)	3.60 (1.4-5.75)	NS
Insoluble NSP (g)	0.55 (0-5.48)	2.00 (0.9-2.8)	NS
Soluble NSP (g)	0.35 (0-4.48)	1.50 (0.55-2.6)	NS
Lignin (g)	0.05 (0-0.38)	0.20 (0.1-0.3)	NS
Total dietary fibre (g)	17.0 (9.23-24.6)	12.2 (8.85-15.8)	NS
Insoluble dietary fibre (g)	0.60 (0-6.1)	2.30 (0.95-3.2)	NS
Soluble dietary fibre (g)	0.35 (0-4.48)	1.50 (0.55-2.6)	NS
Ash (g)	7.75 (3.8-13.8)	6.10 (4.6-12.5)	NS
Moisture (g)	623 (340-1073)	789 (628-1030)	NS
**Minerals – Absolute intakes**			
Ca (mg)	195 (106-382)	132 (85-187)	NS
Fe (mg)	11.3 (6.98-13.5)	10.9 (7.4-12.5)	NS
Haem iron (mg)	0.05 (0-0.63)	0.40 (0-0.55)	NS
Non-haem iron (mg)	1.95 (0.15-4.55)	3.30 (2.25-4.25)	NS
Mg (mg)	280 (116-347)	168 (135-224)	NS
P (mg)	723 (418-855)	573 (420-731)	NS
K (mg)	1838 (910-2858)	1217 (925-1474)	NS
Na (mg)	2126 (1009-3923)	1608 (1010-3360)	NS
Cl (mg)	96.5 (30-406)	397 (245-567)	0.02
Zn (mg)	6.95 (5.16-9.53)	9.46 (7.81-11.97)	NS
Cu (mg)	1.11 (0.48-1.27)	0.74 (0.5-0.96)	NS
Cr (mcg)	10.4 (0-47.03)	31.00 (25.3-49.8)	0.05
Se (mcg)	7.45 (4.38-20.3)	30.5 (24.9-43.5)	<.001
Mn (mcg)	1730 (716-2414)	991 (801-1782)	NS
I (mcg)	91.0 (25.3-168)	92.0 (63.5-189)	NS
B (mcg)	71.5 (0-11)	438 (169-624)	NS
F (mcg)	63.5 (0.75-153)	111 (93-141)	NS
Si (mcg)	0.00 (0-842)	1776 (284-3838)	0.01

**Vitamins – Absolute intakes**			
Vitamin A (RE) (mcg)	423 (330-690)	430 (301-705)	NS
Retinol (mcg)	1.50 (0-73.75)	60.0 (33-75.5)	NS
Total carotenoids (mcg)	745 (0-3408)	384 (162-1434)	NS
β-Carotene (mcg)	627 (0-3368)	281 (156-1265)	NS
α-Carotene (mcg)	0.00 (0-36.3)	104 (0-123)	NS
Cryptoxanthin (mcg)	5.00 (0-39.3)	16.0 (1-23.5)	NS
Thiamin (mg)	1.06 (0.64-1.40)	1.08 (0.69-1.41)	NS
Riboflavin (mg)	0.51 (0.32-1.26)	0.76 (0.65-0.97)	NS
Niacin (mg)	14.6 (9.03-20.28)	16.3 (9.8-19.6)	NS
Vitamin B6 (mg)	2.36 (1.37-5.19)	3.13 (1.66-3.723)	NS
Folate (mcg)	212 (180-383)	278 (207-312)	NS
Vitamin B12 (mcg)	1.65 (0.23-2.45)	1.80 (1.6-3.2)	NS
Pantothenate (mg)	2.33 (2.08-3.02)	2.95 (2.27-4.82)	NS
Biotin (mcg)	14.5 (10.1-27.8)	25.4 (20.8-34.7)	NS
Vitamin C (mg)	31.5 (2.25-91.5)	22.0 (10.5-62.5)	NS
Vitamin D (mcg)	0.86 (0.04-2.08)	6.45 (4.20-8.46)	0.002
Vitamin E (mg)	5.24 (3.32-11.7)	7.91 (4.73-13.3)	NS
α-Tocopherol (mg)	0.38 (0-3.72)	6.08 (3.44-8.67)	0.005
β-Tocopherol (mg)	0.00 (0-0.11)	0.20 (0.12-0.31)	0.002
δ-Tocopherol (mg)	0.00 (0-0.02)	0.11 (0.11-0.22)	<.001
γ-Tocopherol (mg)	0.00 (0-0.21)	0.49 (0.45-0.83)	0.004
α-Tocotrienol (mg)	0.00 (0-0.06)	0.07 (0.07-0.15)	0.005
β-Tocotrienol (mg)	0.00 (0-0.02)	0.05 (0.03-0.06)	0.005
δ-Tocotrienol (mg)	0.00 (0-0)	0.00 (0-0)	-
γ-Tocotrienol (mg)	0.00 (0-0.02)	0.01 (0-0.02)	NS
Lycopene (mcg)	0.00 (0-133)	0.00 (0-2.5)	NS
Lutein (mcg)	38.0 (0-7900)	51.0 (9-140)	NS
Vitamin K (mcg)	23.8 (0.05-403)	19.8 (8.9-57.2)	NS
**Fatty acids (FA) and cholesterol – Absolute intakes**			
Saturated FA (g)	9.31 (5.67-14.9)	8.95 (6.34-13.2)	NS
Mono-unsaturated FA (g)	11.8 (7.01-19.6)	11.8 (7.90-16.4)	NS
Polyunsaturated FA (g)	6.74 (2.74-37.4)	7.15 (5.36-16.0)	NS
Single trans FA (g)	0.00 (0-0)	0.00 (0-0)	-
Double trans FA (g)	0.00 (0-0)	0.00 (0-0)	-
Total trans FA (g)	0.13 (0-1.27)	0.19 (0.03-0.34)	NS
Cholesterol (mg)	65.5 (30-130)	324 (259-461)	<.0001
C4:0 (g)	0.00 (0-0.11)	0.00 (0-0)	NS
C6:0 (g)	0.00 (0-0.07)	0.00 (0-0)	NS
C8:0 (g)	0.00 (0-0.04)	0.00 (0-0.02)	NS
C10:0 (g)	0.01 (0-0.09)	0.02 (0-0.05)	NS
C12:0 (g)	0.04 (0.01-0.21)	0.04 (0.01-0.13)	NS
C13:0 (g)	0.00 (0-0)	0.00 (0-0)	-
C14:0 (g)	0.36 (0.06-0.94)	0.27 (0.18-0.45)	NS
C15:0 (g)	0.00 (0-0)	0.02 (0.01-0.02)	<.001
C16:0 (g)	5.10 (3.04 - 8.57)	4.81 (3.68-7.16)	NS
C17:0 (g)	0.00 (0-0.01)	0.01 (0-0.01)	NS
C18:0 (g)	2.25 (1.36-3.23)	2.25 (1.9-3.72)	NS
C20:0 (g)	0.09 (0.02-0.20)	0.05 (0.02-0.1)	NS
C21:0 (g)	0.00 (0-0)	0.00 (0-0)	-

C22:0 (g)	0.05 (0-0.25)	0.04 (0.02-0.15)	NS
C23:0 (g)	0.00 (0-0)	0.00 (0-0)	-
C24:0 (g)	0.02 (0-0.10)	0.04 (0.02-0.10)	NS
C10:1 (g)	0.00 (0-0)	0.00 (0-0)	-
C12:1 (g)	0.00 (0-0)	0.00 (0-0)	-
C14:1 (g)	0.00 (0-0.04)	0.01 (0-0.02)	NS
C15:1 (g)	0.00 (0-0)	0.00 (0-0)	-
C16:1 (g)	0.33 (0.19-0.53)	0.60 (0.48-0.97)	0.01
C17:1 (g)	0.00 (0-0)	0.00 (0-0)	NS
C18:1 (g)	10.49 (5.57-18.4)	10.09 (6.54-14.5)	NS
C20:1 (g)	0.10 (0.03-0.25)	0.04 (0.02-0.11)	NS
C22:1 (g)	0.04 (0-0.21)	0.00 (0-0.03)	NS
C23:1 (g)	0.00 (0-0)	0.00 (0-0)	-
C24:1 (g)	0.00 (0-0)	0.00 (0-0)	-
C18:2 (g)	5.71 (1.42-35.7)	5.54 (4.09-12.3)	NS
C18:3 (g)	0.26 (0.11-0.99)	0.21 (0.14-0.4)	NS
C18:4 (g)	0.00 (0-0.005)	0.00 (0-0)	NS
C20:2 (g)	0.00 (0-0.01)	0.04 (0.03-0.06)	0.001
C20:4 (g)	0.04 (0-0.07)	0.07 (0.04-0.1)	NS
C20:5 (g)	0.02 (0-0.04)	0.03 (0.02-0.04)	NS
C22:2 (g)	0.00 (0-0.003)	0.01 (0-0.01)	0.03
C22:3 (g)	0.00 (0-0)	0.00 (0-0)	-
C22:4 (g)	0.00 (0-0)	0.00 (0-0)	-
C22:5 (g)	0.00 (0-0.01)	0.00 (0-0.01)	NS
C22:6 (g)	0.03 (0-0.07)	0.07 (0.04-0.08)	NS
C24:6 (g)	0.00 (0-0)	0.00 (0-0)	-
C20:3 (g)	0.00 (0-0.01)	0.00 (0-0.01)	NS
**Amino acids – Absolute intakes**			
Isoleucine (g)	1.46 (0.74-2.10)	1.41 (1.16-2.16)	NS
Leucine (g)	2.76 (1.38-4.08)	2.78 (2.14-3.88)	NS
Lysine (g)	1.72 (0.98-2.90)	2.46 (1.55-3.195)	NS
Methionine (g)	0.83 (0.41-0.96)	0.92 (0.65-1.19)	NS
Phenylalanine (g)	1.59 (0.79-2.39)	1.59 (1.21-2.36)	NS
Threonine (g)	1.31 (0.70-1.92)	1.43 (1.07-1.98)	NS
Tryptophan (g)	0.46 (0.27-0.63)	0.54 (0.38-0.71)	NS
Valine (g)	1.72 (0.85-2.47)	1.68 (1.36-2.49)	NS
Arginine (g)	1.69 (1.13-2.83)	2.23 (1.53-3.27)	NS
Histidine (g)	0.88 (0.41-1.24)	0.93 (0.73-1.29)	NS
Cystine (g)	0.33 (0.20-0.48)	0.64 (0.53-0.81)	<.001
Tyrosine (g)	0.69 (0.29-1.02)	1.06 (0.73-1.40)	0.03
Alanine (g)	0.85 (0.35-1.60)	1.59 (1.11-2.23)	0.02
Aspartic acid (g)	1.45 (0.80-2.69)	2.90 (1.78-3.53)	0.04
Glutamic acid (g)	4.76 (3.04-5.91)	5.50 (3.31-8.31)	NS
Glycine (g)	0.73 (0.36-1.44)	1.33 (0.93-2.00)	0.03
Proline (g)	1.43 (0.89-2.01)	1.74 (1.09-2.58)	NS
Serine (g)	0.98 (0.46-1.59)	1.65 (1.33-2.02)	0.01
Hydroxyproline (g)	0.02 (0-0.18)	0.09 (0-0.17)	NS
Alcohol (g)	0.00 (0-0)	0.00 (0-0)	-
Phytate (mg)	155 (6-192)	192 (99.5-225)	NS
Malic acid (mg)	38.0 (0-879)	305 (14.5-612.5)	NS

Citric acid (mg)	276.50 (0-1275)	221 (77-508)	NS
Tartaric acid (mg)	0.00 (0-0)	0.00 (0-0)	NS
Oxalic acid (mg)	6.00 (0-384)	11.0 (0-37.5)	NS
Caffeine (mg)	0.00 (0-0)	0.00 (0-0)	-
Tannins (mg)	0.00 (0-0)	0.00 (0-0)	-
**Energy – %**			
% Energy - Protein	0.13 (0.08-0.18)	0.15 (0.13-0.18)	NS
% Energy - Fat	0.20 (0.13-0.40)	0.25 (0.21-0.31)	NS
% Energy - Saturated SFA	0.06 (0.04-0.07)	0.06 (0.05-0.09)	NS
% Energy - Mono-unsaturated FA	0.07 (0.05-0.12)	0.09 (0.08-0.11)	NS
% Energy – Polyunsaturated FA	0.04 (0.02-0.16)	0.06 (0.04-0.09)	NS
% Energy - Carbohydrate	0.64 (0.52-0.70)	0.59 (0.51-0.65)	NS

Data from mother-infant dyads that attended follow up are presented as median (IQR) and p values are from univariate analysis (Kruskal-Wallis/Wilcoxon test for non-parametric data; ANOVA for normal data with equal variance). Iodised salt was assumed to be consumed by all mothers, as the majority of salt consumed in South Africa is iodised (61).

Supplementary table S4. Maternal nutrient intake from one 24-hour dietary recall for mothers who report experiencing food insecurity compared to those who do not experience food insecurity.

	**Do you worry about food runout?**		
	**NO**	**YES**	**p value**
	n=14	n=17	
**Dietary diversity score (/9)**	4.50 (4.00, 6.00)	4.00 (3.00, 5.50)	NS
**Dietary diversity score <4 (n)**	2	5	NS
**Absolute intakes**			
Macronutrients			
Moisture (g)	868 (629, 1029)	670 (507, 1092)	NS
Energy (kJ)	5601 (4792, 6855)	5204 (3264, 8103)	NS
Nitrogen (g)	7.15 (5.55, 8.82)	4.63 (3.93, 6.57)	NS
Total protein (g)	51.4 (43.8, 63.2)	37.9 (26.3, 53.8)	NS
Plant protein (g)	19.6 (17.1, 26.0)	22.3 (11.1, 37.5)	NS
Animal protein (g)	29.0 (19.3, 39.0)	16.3 (3.85, 30.0)	0.03
Total fat (g)	36.5 (30.0, 51.8)	25.8 (20.6, 65.1)	NS
Carbohydrates, avail. (g)	162 (159, 200)	161 (97.0, 277)	NS
Starch (g)	5.6 (0.18, 10.3)	0.00 (0.00, 8.1)	NS
Glucose (g)	3.95 (2.40, 5.00)	1.80 (0.00, 3.55)	0.04
Fructose (g)	6.50 (0.90, 7.45)	0.70 (0.00, 6.30)	NS
Sucrose (g)	17.7 (12.0, 32.9)	12.0 (1.70, 23.6)	NS
Maltose (g)[1]		0.1	
Galactose[2]			
Lactose (g)	0.00 (0.00, 2.10)	0.00 (0.00, 0.05)	NS
Total sugars (g)	30.3 (23.4, 41.7)	15.8 (8.05, 34.5)	NS
Added sugar (g)	25.6 (12.0, 41.0)	8.00 (0.50, 18.8)	0.01
Total dietary fibre (g)	13.4 (11.0, 16.7)	12.2 (6.70, 21.1)	NS
Insoluble dietary fibre (g)	2.75 (1.33, 3.60)	1.40 (0.00, 3.45)	NS
Soluble dietary fibre (g)	1.85 (1.05, 2.70)	0.80 (0.00, 3.40)	NS
Ash (g)	6.05 (4.65, 6.53)	9.30 (4.30, 14.8)	NS
Non-starch polysaccharides (g)	4.20 (2.13, 5.98)	2.10 (0.00, 6.45)	NS
Insoluble NSP (g)	2.25 (1.15, 3.03)	1.40 (0.00, 3.05)	NS
Soluble NSP (g)	1.85 (1.05, 2.70)	0.80 (0.00, 3.40)	NS
Lignin (g)	0.20 (0.10, 0.30)	0.10 (0.00, 0.30)	NS
Amino acids			
Isoleucine (g)	1.78 (1.34, 2.26)	1.24 (0.80, 1.83)	NS
Leucine (g)	3.48 (2.68, 4.06)	2.18 (1.51, 3.34)	NS
lysine (g)	2.59 (1.84, 3.67)	1.52 (1.04, 2.65)	NS
Methionine (g)	0.96 (0.88, 1.17)	0.65 (0.47 (0.91)	0.01
Phenylalanine (g)	2.01 (1.53, 2.35)	1.32 (0.90, 2.11)	NS
Threonine (g)	1.73 (1.33, 2.10)	1.08 (0.76, 1.71)	NS
Tryptophan (g)	0.61 (0.53, 0.71)	0.38 (0.30, 0.57)	0.02
Valine (g)	212 (1.67, 2.45)	1.38 (0.98, 2.23)	NS
Arginine (g)	2.53 (1.86, 3.36)	1.48 (1.18, 2.77)	NS
Histidine (g)	1.14 (0.85, 1.37)	0.73 (0.44, 1.27)	0.05
Cystine (g)	0.62 (0.53, 0.87)	0.48 (0.31, 0.66)	0.02
Tyrosine (g)	1.12 (0.87, 1.44)	0.79 (0.46, 1.06)	NS
Alanine (g)	1.74 (1.28, 2.28)	1.16 (0.67, 1.65)	0.04

Aspartic acid (g)	3.01 (2.24, 3.71)	1.87 (1.12, 2.92)	NS
Glutamic acid (g)	6.24 (4.72, 8.31)	4.43 (1.96, 5.83)	NS
Glycine (g)	1.47 (1.03, 2.10)	1.02 (0.52, 1.47)	NS
Proline (g)	2.00 (1.47, 2.63)	1.36 (0.56, 1.87)	0.05
Serine (g)	1.65 (1.50, 2.14)	1.20 (0.86, 1.67)	0.03
Hydroxyproline (g)	0.10 (0.06, 0.22)	0.00 (0.00, 0.11)	NS
Fatty acids and cholesterol			
Saturated FA (g)	9.55 (8.56, 15.2)	7.88 (5.74, 11.4)	NS
Mono-unsaturated FA (g)	12.5 (10.5, 15.8)	10.9 (7.19, 21.8)	NS
Polyunsaturated FA (g)	8.70 (6.26, 15.3)	5.46 (3.37, 20.2)	NS
Single trans FA (g)[2]			
Double trans FA (g)[2]			
Total trans FA (g)	0.20 (0.16, 0.82)	0.06 (0.00, 0.35)	NS
Cholesterol (mg)	291 (236, 544)	117 (33.0, 420)	0.04
**Median intake (%) of TULs by mothers**			
Minerals			
Calcium (Ca)	5.88 (4.56, 9.55)	4.52 (2.76, 10.5)	NS
Iron (Fe)	25.6 (21.7, 27.1)	18.2 (12.8, 31.6)	NS
Magnesium (Mg)	52.1 (44.6, 71.4)	57.1 (30.0, 97.0)	NS
Phosphorus (P)	16.7 (12.9, 19.9)	12.1 (9.14, 18.9)	NS
Sodium (Na)	67.5 (48.0, 91.7)	127 (42.5, 210)	NS
Zinc (Zn)	22.3 (20.1, 29.6)	19.6 (14.7, 27.1)	NS
Copper (Cu)	8.15 (6.35, 10.6)	6.90 (3.95, 12.1)	NS
Selenium (Se)	7.56 (5.41, 11.6)	6.18 (1.86, 9.73)	NS
Manganese (Mn)	12.8 (8.46, 18.2)	12.7 (5.59, 18.9)	NS
Iodine (I)	7.82 (3.73, 12.8)	13.6 (4.59, 19.1)	NS
Vitamins			
Vitamin A	14.2 (11.2, 18.8)	14.1 (8.57, 26.9)	NS
Niacin	47.4 (42.0, 55.6)	40.0 (24.4, 60.6)	NS
Vitamin B6	3.20 (2.74, 3.68)	2.14 (1.22, 4.17)	NS
Folate	27.8 (21.3, 29.6)	24.1 (19.8, 48.6)	NS
Vitamin C	1.28 (0.90, 2.45)	0.60 (0.08, 6.38)	NS
Vitamin D	4.73 (4.15, 8.76)	1.39 (0.39, 7.86)	NS
Vitamin E	0.73 (0.49, 1.21)	0.68 (0.41, 1.30)	NS
	**Do you experience food runout?**		
	**NO**	**YES**	**p value**
	n=16	n=15	
**Dietary diversity score (/9)**	5.00 (4.00, 6.00)	3.00 (4.00, 5.00)	NS
**Dietary diversity score <4 (n)**	2	5	NS
**Absolute intakes**			
Macronutrients			
Moisture (g)	868 (620, 1030)	670 (409, 1011)	NS
Energy (kJ)	5601 (4659, 7004)	5204 (3247, 8339)	NS
Nitrogen (g)	7.15 (5.68, 9.29)	4.31 (3.87, 6.10)	0.02
Total protein (g)	51.4 (42.5, 68.7)	32.9 (26.1, 44.7)	0.03

Plant protein (g)	19.6 (15.8, 27.3)	22.3 (11.5, 34.9)	NS
Animal protein (g)	29.0 (19.7, 38.0)	15.4 (3.30, 21.4)	0.01
Total fat (g)	35.0 (29.9, 45.8)	25.8 (19.6, 69.0)	NS
Carbohydrates, avail. (g)	162 (159, 234)	161 (93.8, 280)	NS
Starch (g)	5.60 (0.13, 10.1)	0.00 (0.00, 5.40)	NS
Glucose (g)	3.95 (2.75, 5.60)	1.20 (0.00, 3.40)	0.01
Fructose (g)	6.50 (1.20, 8.15)	0.40 (0.00, 6.10)	0.01
Sucrose (g)	19.1 (12.4, 30.1)	8.40 (1.00, 17.9)	0.03
Maltose (g)[1]		0.1	
Galactose[2]			
Lactose (g)	0.00 (0.00, 1.60)	0.00 (0.00, 0.10)	NS
Total sugars (g)	31.2 (24.3, 42.5)	13.1 (7.00, 30.9)	0.02
Added sugar (g)	17.6 (12.0, 40.5)	8.00 (0.30, 21.0)	0.04
Total dietary fibre (g)	13.4 (10.9, 17.2)	12.2 (6.20, 18.4)	NS
Insoluble dietary fibre (g)	3.15 (1.68, 4.08)	1.20 (0.00, 3.20)	0.04
Soluble dietary fibre (g)	2.20 (1.30, 2.70)	0.70 (0.00, 2.90)	NS
Ash (g)	6.05 (4.55, 6.58)	10.5 (4.20, 14.8)	NS
Non-starch polysaccharides (g)	4.85 (2.68, 6.33)	1.80 (0.00, 5.90)	NS
Insoluble NSP (g)	2.60 (1.43, 3.50)	1.10 (0.00, 2.80)	NS
Soluble NSP (g)	2.20 (1.30, 2.70)	0.70 (0.00, 2.90)	NS
Lignin (g)	0.25 (0.13, 0.38)	0.00 (0.00, 0.20)	0.01
Amino acids			
Isoleucine (g)	1.78 (1.38, 2.43)	1.08 (0.79, 1.47)	0.05
Leucine (g)	3.48 (2.77, 4.47)	1.95 (1.47, 2.75)	0.03
lysine (g)	2.59 (1.99, 3.80)	1.23 (1.03, 2.57)	0.03
Methionine (g)	0.96 (0.88, 1.13)	0.61 (0.45, 0.85)	0.002
Phenylalanine (g)	2.01 (1.55, 2.51)	1.22 (0.80, 1.69)	0.04
Threonine (g)	1.73 (1.38, 2.22)	0.95 (0.74, 1.43)	0.02
Tryptophan (g)	0.61 (0.53, 0.76)	0.34 (0.30, 0.51)	0.003
Valine (g)	2.12 (1.68, 2.56)	1.24 (0.87, 1.75)	0.04
Arginine (g)	2.53 (1.96, 3.57)	1.48 (1.17, 2.12)	0.03
Histidine (g)	1.14 (0.89, 1.51)	0.64 (0.42, 1.26)	0.01
Cystine (g)	0.62 (0.52, 0.81)	0.48 (0.30, 0.62)	0.02
Tyrosine (g)	1.14 (0.94, 1.42)	0.75 (0.46, 0.90)	0.03
Alanine (g)	1.75 (1.37, 2.29)	0.92 (0.66, 1.46)	0.006
Aspartic acid (g)	3.01 (2.46, 3.74)	1.47 (1.04, 2.90)	0.04
Glutamic acid (g)	5.85 (5.25, 8.12)	4.33 (1.70, 5.78)	NS
Glycine (g)	1.52 (1.13, 2.14)	0.84 (0.42, 1.30)	0.009
Proline (g)	1.85 (1.58, 2.59)	1.28 (0.51, 1.92)	0.03
Serine (g)	1.68 (1.52, 2.02)	1.09 (0.85, 1.48)	0.01
Hydroxyproline (g)	0.11 (0.06, 0.26)	0.00 (0.00, 0.07)	0.005
Fatty acids and cholesterol			
Saturated FA (g)	9.68 (8.88, 14.7)	6.80 (5.69, 12.9)	0.03
Mono-unsaturated FA (g)	11.7 (10.4, 15.0)	12.0 (6.86, 25.7)	NS
Polyunsaturated FA (g)	7.67 (5.87, 13.9)	5.47 (3.88, 22.7)	NS
Single trans FA (g)[2]			
Double trans FA (g)[2]			
Total trans FA (g)	0.21 (0.16, 0.75)	0.04 (0.00, 0.20)	0.03

Cholesterol (mg)	291 (191, 524)	112 (26.0, 416)	0.03
**Median intake (%) of TULs by mothers**			
Minerals			
Calcium (Ca)	5.88 (4.56, 9.90)	4.52 (2.72, 9.56)	NS
Iron (Fe)	25.6 (21.0, 27.4)	18.2 (12.0, 30.2)	NS
Magnesium (Mg)	52.1 (43.1, 74.6)	57.1 (30.3, 95.1)	NS
Phosphorus (P)	16.7 (12.8, 20.7)	12.1 (8.65, 18.5)	NS
Sodium (Na)	61.9 (47.6, 84.9)	132 (51.2, 221)	NS
Zinc (Zn)	23.3 (20.2, 30.3)	17.7 (14.4, 24.2)	NS
Copper (Cu)	8.15 (6.43, 11.7)	6.90 (3.90, 11.0)	NS
Selenium (Se)	7.63 (5.91, 11.2)	5.58 (1.80, 7.78)	NS
Manganese (Mn)	12.8 (8.77, 19.3)	12.7 (5.36, 16.7)	NS
Iodine (I)	7.67 (3.73, 11.2)	14.0 (5.09, 19.6)	NS
Vitamins			
Vitamin A	14.2 (10.5, 20.9)	14.1 (8.54, 25.2)	NS
Niacin	47.4 (41.4, 55.9)	40.0 (23.4, 55.7)	NS
Vitamin B6	3.20 (2.72, 3.89)	1.89 (1.16, 3.80)	NS
Folate	27.8 (20.0, 30.1)	24.1 (20.5, 47.6)	NS
Vitamin C	1.34 (0.99, 2.55)	0.55 (0.00, 6.25)	NS
Vitamin D	4.73 (4.14, 8.60)	1.39 (0.05, 7.86)	NS
Vitamin E	0.73 (0.49, 1.16)	0.68 (0.39, 1.46)	NS
	**Are you able to afford balanced meals?**		
	**YES**	**NO**	**p value**
	n=9	n=22	
**Dietary diversity score (/9)**	4.00 (3.50, 5.50)	4.00 (3.75, 6.00)	NS
**Dietary diversity score <4 (n)**	2	5	NS
**Absolute intakes**			
Macronutrients			
Moisture (g)	789 (614, 1037)	806 (564, 1016)	NS
Energy (kJ)	5691 (4519, 7046)	5358 (3538, 7505)	NS
Nitrogen (g)	6.94 (5.00, 9.53)	5.52 (4.03, 7.37)	NS
Total protein (g)	53.3 (41.5, 67.7)	40.9 (27.5, 54.6)	NS
Plant protein (g)	19.9 (17.0, 33.1)	20.6 (11.6, 34.0)	NS
Animal protein (g)	28.5 (19.0, 38.5)	18.9 (9.65, 33.2)	NS
Total fat (g)	37.5 (25.1, 57.2)	31.0 (21.9, 63.1)	NS
Carbohydrates, avail. (g)	165 (158, 229)	160 (106, 259)	NS
Starch (g)	5.60 (0.05, 6.80)	0.20 (0.00, 12.5)	NS
Glucose (g)	3.80 (2.50, 5.25)	2.55 (0.30, 4.55)	NS
Fructose (g)	6.50 (1.90, 7.75)	1.10 (0.00, 6.65)	NS
Sucrose (g)	20.4 (16.3, 28.1)	12.0 (3.98, 23.9)	NS
Maltose (g)[1]		0.1	
Galactose[2]			
Lactose (g)	0.00 (0.00, 2.40)	0.00 (0.00, 0.15)	NS
Total sugars (g)	32.0 (29.6, 39.5)	20.8 (9.10, 37.1)	NS
Added sugar (g)	13.4 (10.5, 25.6)	12.0 (3.70, 39.5)	NS
Total dietary fibre (g)	14.5 (11.6, 16.3)	12.3 (8.10, 17.9)	NS

Insoluble dietary fibre (g)	3.10 (1.35, 3.90)	1.75 (0.00, 3.33)	NS
Soluble dietary fibre (g)	2.20 (0.85, 2.75)	1.30 (0.00, 2.75)	NS
Ash (g)	6.10 (4.25, 6.55)	6.15 (4.63, 14.6)	NS
Non-starch polysaccharides (g)	4.90 (2.00, 6.25)	2.75 (0.00, 6.05)	NS
Insoluble NSP (g)	2.50 (1.05, 3.30)	1.45 (0.00, 2.98)	NS
Soluble NSP (g)	2.20 (0.85, 2.75)	0.00 (1.30, 2.75)	NS
Lignin (g)	0.30 (0.20, 0.40)	0.10 (0.00, 0.23)	NS
Amino acids			
Isoleucine (g)	1.77 (1.26, 2.55)	1.50 (0.89, 1.95)	NS
Leucine (g)	3.36 (2.60, 2.46)	2.47 (1.58, 3.71)	NS
lysine (g)	2.71 (1.81, 4.05)	1.74 (1.06, 2.65)	NS
Methionine (g)	1.06 (0.93, 2.44)	1.01 (0.78, 1.64)	NS
Phenylalanine (g)	1.96 (1.42, 2.43)	1.51 (1.00, 2.22)	NS
Threonine (g)	1.69 (1.26, 2.23)	1.25 (0.79, 1.81)	NS
Tryptophan (g)	0.59 (0.48, 0.77)	0.42 (0.31, 0.63)	NS
Valine (g)	2.01 (1.53, 2.58)	1.57 (1.12, 2.38)	NS
Arginine (g)	2.44 (1.79, 3.49)	1.80 (1.28, 2.68)	NS
Histidine (g)	1.16 (0.82, 1.51)	0.76 (0.50, 1.20)	NS
Cystine (g)	0.60 (0.48, 0.78)	0.52 (0.34, 0.72)	NS
Tyrosine (g)	1.09 (0.80, 1.40)	0.85 (0.49, 1.10)	NS
Alanine (g)	1.76 (1.20, 2.32)	1.24 (0.73, 1.73)	NS
Aspartic acid (g)	2.93 (2.06, 3.90)	1.87 (1.30, 3.02)	NS
Glutamic acid (g)	5.71 (4.26, 7.54)	4.58 (2.39, 6.46)	NS
Glycine (g)	1.49 (1.06, 2.13)	1.04 (0.63, 1.48)	NS
Proline (g)	1.81 (1.36, 2.35)	1.38 (0.78, 2.36)	NS
Serine (g)	1.65 (1.29, 2.02)	1.43 (0.91, 1.71)	NS
Hydroxyproline (g)	0.11 (0.07, 0.24)	0.00 (0.00, 0.11)	0.04
Fatty acids and cholesterol			
Saturated FA (g)	9.77 (8.33, 19.5)	7.96 (5.98, 12.1)	NS
Mono-unsaturated FA (g)	11.8 (8.26, 16.4)	11.5 (7.6, 21.8)	NS
Polyunsaturated FA (g)	9.40 (4.85, 15.5)	6.34 (4.49, 18.4)	NS
Single trans FA (g)[2]			
Double trans FA (g)[2]			
Total trans FA (g)	0.73 (0.16, 1.00)	0.06 (0.00, 0.21)	0.03
Cholesterol (mg)	269 (213, 326)	261 (45.5, 446)	NS
**Median intake (%) of TULs by mothers**			
Minerals			
Calcium (Ca)	7.04 (4.90, 11.7)	4.76 (3.01, 8.33)	NS
Iron (Fe)	25.8 (213, 30.0)	24.2 (14.6, 28.3)	NS
Magnesium (Mg)	54.0 (46.4, 78.7)	52.1 (30.7, 88.6)	NS
Phosphorus (P)	15.2 (12.9, 21.2)	14.6 (9.83, 18.9)	NS
Sodium (Na)	65.1 (48.4, 95.0)	73.6 (45.0, 181)	NS
Zinc (Zn)	20.9 (20.2, 36.8)	21.0 (15.2, 26.6)	NS
Copper (Cu)	8.60 (6.30, 10.7)	7.15 (4.45, 11.4)	NS
Selenium (Se)	7.50 (6.03, 8.66)	6.45 (2.73, 9.89)	NS
Manganese (Mn)	14.7 (8.84, 27.1)	11.5 (6.52, 16.9)	NS
Iodine (I)	8.18 (3.59, 13.6)	8.36 (4.84, 18.4)	NS

Vitamins			
Vitamin A	12.2 (10.2, 16.1)	14.7 (8.95, 24.9)	NS
Niacin	46.6 (41.7, 54.0)	42.1 (24.9, 58.1)	NS
Vitamin B6	3.26 (2.80, 5.09)	2.61 (1.37, 3.69)	NS
Folate	27.8 (22.6, 29.9)	25.0 (19.3, 37.1)	NS
Vitamin C	2.40 (0.85, 3.90)	1.13 (0.23, 3.59)	NS
Vitamin D	4.72 (4.20, 6.22)	4.12 (0.72, 8.06)	NS
Vitamin E	0.80 (0.51, 1.13)	0.62 (0.42, 1.50)	NS

Data from mother-infant dyads that attended follow up are presented as mean ± SD or median (IQR) and p values are from univariate analysis (Kruskal-Wallis/Wilcoxon test for non-parametric data; ANOVA for normal data with equal variance). Dietary diversity score was calculated using nine food groups, only counting each food groups once (39, 40). Percent intake of TULs were calculated using the Institute of Medicine's TULs for minerals and vitamins for lactating women 14-18, 19-30 or 31-50 years of age (37). Iodised salt was assumed to be consumed by all mothers, as the majority of salt consumed in South Africa is iodised (61). TULs = Tolerable upper levels. [1]Only one mother consumed maltose. [2]None consumed by mothers.
